# Genome-Scale Investigation of the Regulation of *azoR* Expression in *Escherichia coli* Using Computational Analysis and Transposon Mutagenesis

**DOI:** 10.1007/s00248-024-02380-5

**Published:** 2024-05-01

**Authors:** Mona A. Salem, Hanzada T. Nour El-Din, Abdelgawad M. Hashem, Ramy K. Aziz

**Affiliations:** 1 Department of Microbiology, Faculty of Pharmacy, The British University in Egypt (BUE), 11837 El-Sherouk City, Egypt; 2 Department of Microbiology and Immunology, Faculty of Pharmacy, Cairo University, Qasr El-Ainy St, 11562 Cairo, Egypt; 3 Center for Genome and Microbiome Research, Cairo University, 11562 Cairo, Egypt; 4 Microbiology and Immunology Research Program, Children’s Cancer Hospital Egypt, 57357, 11617 Cairo, Egypt

**Keywords:** *azoR*, Azoreductases, Gene Expression Omnibus (GEO), Methyl red decolorization, Random transposon mutagenesis, Regulation

## Abstract

Bacterial azoreductases are enzymes that catalyze the reduction of ingested or industrial azo dyes. Although azoreductase genes have been well identified and characterized, the regulation of their expression has not been systematically investigated. To determine how different factors affect the expression of *azoR*, we extracted and analyzed transcriptional data from the Gene Expression Omnibus (GEO) resource, then confirmed computational predictions by quantitative reverse transcription polymerase chain reaction (qRT-PCR). Results showed that *azoR* expression was lower with higher glucose concentration, agitation speed, and incubation temperature, but higher at higher culture densities. Co-expression and clustering analysis indicated ten genes with similar expression patterns to a*zoR*: *melA*, *tpx*, *yhbW*, *yciK*, *fdnG*, *fpr*, *nfsA*, *nfsB*, *rutF*, and *chrR* (*yieF*). In parallel, constructing a random transposon library in *E. coli* K-12 and screening 4320 of its colonies for altered methyl red (MR)-decolorizing activity identified another set of seven genes potentially involved in *azoR* regulation. Among these genes, *arsC*, *relA*, *plsY*, and *trmM* were confirmed as potential *azoR* regulators based on the phenotypic decolorization activity of their transposon mutants, and the expression of *arsC* and *relA* was confirmed, by qRT-PCR, to significantly increase in *E. coli* K-12 in response to different MR concentrations. Finally, the significant decrease in *azoR* transcription upon transposon insertion in *arsC* and *relA* (as compared to its expression in wild-type *E. coli*) suggests their probable involvement in *azoR* regulation. In conclusion, combining in silico analysis and random transposon mutagenesis suggested a set of potential regulators of *azoR* in *E. coli*.

**Supplementary Information:**

The online version contains supplementary material available at 10.1007/s00248-024-02380-5.

## Introduction

Azo dyes are among the most commonly used synthetic dyes, with extensive applications in food, pharmaceuticals, cosmetics, and textile industries, among others [1]. They are characterized by the presence of one or more azo groups (– N = N –) that account for their recalcitrant xenobiotic nature [2]. Approximately 15% of the azo dyes used in the textile industry are released into the environment as industrial effluents, which contaminate agricultural crops, decrease oxygen levels and pH in rivers, and disturb the aquatic ecosystem, evoking a significant environmental concern [3]. Additionally, many azo dyes are toxic, and their biotransformation products can be carcinogenic and mutagenic; therefore, they are disapproved for use as food components [4]. Because of this problem, ecofriendly, cost-effective biological systems have been thoroughly studied for their potential application as decolorizing agents in industry [5].

Bacteria and other microorganisms, including yeast and filamentous fungi, exert their degradative effect by secreting a number of enzymes, such as azoreductases, laccases, oxidases, and peroxidases [6]. A number of azoreductases were identified in *Escherichia coli*, two of which, AzoRI and AzoRII, are NAD(P)H dependent azoreductases that degrade Ponceau SX most efficiently, besides Tartrazine, Amaranth, and Orange II [7]. However, the most common azoreductase in *E. coli* is AzoR, an FMN-dependent-NADH azoreductase that follows a ping pong Bi-Bi mechanism of action in two cycles [8]. Other studies showed that some azoreductases, including the *E. coli* AzoR, act as quinone reductases, thus providing resistance to thiol-specific stress [9–11]. In *Pseudomonas aeruginosa*, azoreductases were predicted to be potentially involved in bacterial pathogenicity, and both *azoR1* and *azoR2* genes were required for systemic infection in mice [12].

Although azoreductase activity has been thoroughly studied in several bacteria, only limited research has been conducted on the regulation of its gene expression, particularly in *E. coli*. A pilot study used whole-genome microarrays in *E. coli* K-12 to determine the changes in gene expression during acid red 18 (AR18) decolorization, and found upregulated genes to be related to stress response, metabolism, and cell components. Upon deletion of five upregulated genes, the decolorization rate of AR18 by *E. coli* K-12 decreased but did not completely stop, suggesting the presence of alternative decolorization pathway [13].

The previous data suggest that bacterial azoreductases may have more than one physiological role that is yet to be elucidated to reveal how their homologs in human-associated and environmental isolates react to xenobiotics [10]. Here, we aimed to use two complementary approaches to investigate, on a genome-scale, the regulation of *azoR* in *E. coli*—as a model organism: (i) a computational approach that integrates data from public databases, and (ii) an experimental loss-of-function approach through random transposon mutagenesis, followed by screening and transcriptional analysis. Using those two complementary approaches, we shortlisted a set of four candidate regulators of *azoR* expression and experimentally confirmed the involvement of two of them, *arsC* and *relA*, in the decolorization process of the model dye methyl red.

## Methods

### Bacterial Strains and Culture Conditions

The wild-type (WT) *E. coli* K-12 MG1655 was used for the construction of the transposon library. ∆*azoR*, an *E. coli* K-12 strain with the *azoR* gene knocked out, from the Keio collection [14] was used for optimization of the 96-well plate decolorization assay.

*E. coli* K-12 was regularly grown on Luria Bertani (LB) broth or agar (Conda, Spain) at 37 °C with shaking at 250 rpm unless otherwise specified. For decolorization experiments, *E. coli* WT and mutants were cultivated in mineral salts medium (MSM) containing 4 g K_2_HPO_4_; 4 g KH_2_PO_4_; 2 g (NH_4_)_2_SO_4_; 0.5 g MgSO_4_·7H_2_O; 0.01 g CaCl_2_; 0.01 g FeSO_4_·7H_2_O per liter of distilled water (pH 7 ± 0.2) supplemented with 0.1% yeast. Methyl red (MR) (Alfa-Aesar, Germany) was prepared as a 500 mg/L stock then filter sterilized through a 0.22-µm sterile cellulose acetate membrane filter. Fifty microgram per milliliter kanamycin (Bio Basic, Canada) was used to positively select transposon mutants when necessary.

### Systems Analysis of Transcriptomic Data

#### In Silico *azoR* Expression Analysis

As a first approach to study the regulation of published azoreductase genes in the organisms of interest, microarray data for the *E. coli* azoreductases were mined at the National Center for Biotechnology Information (NCBI)’s GEO database (https://www.ncbi.nlm.nih.gov/geo/) and analyzed.

Briefly, when we searched GEO for “*azoR* and *E. coli*,” 462 samples from 50 datasets of high-throughput gene expression data, recorded as percentile ranks, were collected, and *azoR* transcriptional data was analyzed using R statistical package (R version 4.0.2) to determine conditions under which it is significantly up or downregulated across various research studies not exclusively related to azoreduction. Any sample missing *azoR* expression value was filtered out. The selected 462 samples were those that had expression percentile ranks for the *azoR* gene (among all other measured *E. coli* genes), in addition to at least one experimental condition listed. The studied conditions included the effect of culture media, growth phase, glucose concentration, culture density, incubation temperature, and agitation speed on *azoR* expression.

#### In Vitro Validation of the Conditions Affecting *azoR* Expression in *E. coli*

To confirm the reliability of the analyzed public transcriptome data, we quantified *azoR* expression in *E. coli* K-12 grown under different growth conditions. To test the effect of different glucose concentrations while using it as a sole source of carbon, we used the RNeasy Mini Kit (Qiagen, Germany) to extract RNA from *E. coli* samples grown to mid-log phase in MSM, supplemented with different glucose concentrations of 0.02%, 0.2%, and 0.5%. To test the effects of different OD_600_, incubation temperatures, and agitation speeds, we extracted RNA from *E. coli* samples grown in LB broth to OD_600_ of 0.4, 0.8, and 1.2, as well as *E. coli* samples incubated at two different temperatures (30 °C and 37 °C), and finally, *E. coli* samples incubated at different agitation speeds of 60 and 250 rpm, respectively.

The purity and concentration of RNA were checked by Nanodrop (Quawell, China), then the extracted RNA was treated with DNaseI (New England Biolabs, USA) to ensure the absence of any genomic DNA contamination. Subsequently, we used RevertAid First Strand cDNA Synthesis Kit (Thermo Fisher, USA) for cDNA synthesis, according to the manufacturer’s protocol. The purity and concentration of the prepared cDNA were measured by Nanodrop (Quawell, China). All qRT-PCR experiments were carried out in the StepOnePlus, Real-Time PCR System (Applied Biosystems, USA) with the Maxima Sybr Green qPCR Master Mix (Thermo Scientific, USA). The *azoR* primer pair (Supplementary Table S1) was designed by primer3plus (http://www.bioinformatics.nl/cgi-bin/primer3plus/primer3plus.cgi) and synthesized by Macrogen (Korea). The results were normalized to the *ihfB* reference gene [15]. All assays were performed in triplicates.

The data were analyzed by the delta delta cycle threshold (∆∆Ct) relative quantification method. The data were then presented as fold increase or decrease in terms of the transcriptional level of each sample compared to the control. Statistical analysis between the Cts for each sample was conducted by one-way ANOVA followed by post hoc *t*-test with Tukey’s adjustment for multiple variables (i.e., glucose concentrations and OD_600_). For comparisons involving two variables (i.e., temperature and agitation speed), a paired *t*-test was used. GraphPad Prism 8 (GraphPad Software Tools, Inc., La Jolla, CA, USA) was used for visualizing the data and calculating the *p* values.

### Co-expression Profile and Analysis

Profile neighbors are the top 200 genes with similar expression patterns to the gene of interest in this specific dataset. Profile neighbors’ data for each of the collected 50 datasets were retrieved from the GEO to determine co-expressed genes with *azoR*. Statistical analysis was carried out to determine the most repeated genes, in terms of expression patterns, to *azoR *within all datasets and thus help predict functionally related genes. The maximum number of repetitions was identified, and then a cutoff of genes showing at least half of this number of similar expression pattern repetitions with *azoR* were further processed. Genes (in the form of UniProt accession numbers) were then clustered according to their functional annotations using the Database for Annotation, Visualization and Integrated Discovery (DAVID) [16] (https://david.ncifcrf.gov/).

### Transposon Library Construction and Screening

#### Random Transposon Mutagenesis in *E. coli* and Colony Selection

To determine the genetic elements involved in *azoR* regulation, we constructed an *E. coli* K-12 transposon library as follows. An overnight *E. coli* K-12 culture, grown to an OD_600_ of 0.5, was harvested and made electrocompetent by three rounds of 10% glycerol wash and subsequent centrifugation for 10 min at 1700 ×g and 4 °C. Fifty microliters of the prepared electrocompetent cells were transformed with 1 µl of EZ-Tn5™<KAN-2 > Tnp Transposome (Epicenter, Illumina, USA) by electroporation (Bio-Rad Micropulser, USA). Bacterial cells were recovered for 60 min using 950 µl super optimal broth with catabolite repression (SOC) medium at 37 °C with shaking at 200 rpm. The library was plated on LB agar, supplemented with 50 µg/ml kanamycin, for positive selection of successfully transposed cells. Kanamycin-resistant colonies were picked and arrayed into 96-well plates, and 25% glycerol stocks were prepared in duplicates and stored at −80 °C.

#### Decolorization Activity Screening of the Transposon Library

Decolorization activity of the mutant library was tested by a high-throughput 96-well plate assay as previously described [17], with some modifications that were based on our specific assay optimization. Tests for assay optimization were carried out to identify the conditions that maximize the differences in decolorization activity between the WT and the mutants in terms of wavelength, incubation time and culture media (Supplementary Methods and Supplementary Fig. S1 and S2).

Briefly, sterile, flat-bottomed 96-well plates (SPL, Life Sciences, Korea) were filled with 135 µl MSM supplemented with 0.1% yeast, 50 mg/L dye, and 50 µg/ml kanamycin. Fifteen microliters of an overnight culture of each of the mutants and the WT *E. coli* were added to the wells. Plates were incubated overnight at 30 °C with shaking at 150 rpm. Subsequently, decolorization was measured in a microplate reader (ELx800, Biotek, USA) set with a 450-nm filter. The decolorization ability of each strain, expressed as a percentage, was calculated by the following equation:

$$\mathrm{Decolorization}\;\mathrm{percentage}\;(\%)=\frac{\mathrm{Initial}\;\mathrm{absorbance}-\mathrm{Final}\;\mathrm{absorbance}}{\mathrm{Initial}\;\mathrm{absorbance}}\times100$$ 

Initial and final absorbance correspond to the absorbance measured at 0 and 16 h, respectively.

For ease of determining the cluster or deviation of the mutants, in terms of decolorization activity, from those of the WT strain, the relative mutant decolorization was calculated as follows:

$$\mathrm{Normalized}\;\mathrm{mutant}\;\mathrm{decolorization}=\frac{\mathrm{Mutant}'\mathrm s\;\mathrm{decolorization}\%}{\mathrm{WT}\;\mathrm{decolorization}\;\%}\times100$$ 

High-throughput screening of the decolorization activity of the library facilitated setting a cutoff for mutants showing a normalized mutant decolorization  ≤ 65 or ≥ 110 for further testing. Such mutants were accordingly retested in triplicates by the same 96-well plate assay, before further confirmation in large volumes in 250-ml Erlenmeyer flasks, as previously described [18]. A two-tailed *t*-test was applied to determine the statistical significance between the decolorization percentages of the mutants and the WT.

#### Transposon Insertion Site Determination

Transposon insertion positions for the selected mutants were identified by the previously developed rapid amplification of transposon ends (RATE) PCR which is a single transposon-specific primer-PCR protocol that comprises three rounds of amplification in a single PCR run followed by sequencing the PCR products with a nested primer [19]. GeneAmp^®^ High Fidelity PCR System was used for amplification of the extracted DNA from the chosen mutants in compliance with the manufacturer’s protocol.

The first round of PCR amplification was as follows: initial denaturation of 95 °C for 5 min, followed by 30 cycles of 95 °C/30 s., 55 °C/30 s., and 72 °C/3 min each. The second round comprised 30 cycles of 95 °C/30 s., 30 °C/30 s., and 72 °C/2 min. each. The final round, comprised 33 cycles of 95 °C/30 s., 55 °C/30 s., and 72 °C/2 min each. DNA from the gel bands was subsequently purified by the QIAquick Gel Extraction Kit (Qiagen^®^, Germany). DNA’s purity and concentration were further checked by the Nanodrop device (Quawell, China). Finally, a single nested kanamycin specific primer KAN2-RP1 (Table S1) was used for unidirectional Sanger sequencing of the PCR product. The samples were sequenced by Macrogen, Korea, via Green Tech, Egypt, in a high-throughput Applied Biosystems 3730XL sequencer.

A nucleotide BLAST (BLASTn) [20] of the query sequences against the *E. coli* K-12 substr. MG1655 genome, with default settings, was performed after transposon ends removal and ambiguous base removal by the sequence editor Chromas (software version 2.6.6).

#### Quantifying Gene Expression of Candidate *azoR* Regulators by qRT-PCR

To further investigate the role of four of the sequenced mutants (*∆arsC*, *∆relA*, *∆plsY*, and *∆trmM*) as candidate *azoR* regulators, first, *azoR* expression was compared with that of the four genes of interest in the WT. An overnight culture of *E. coli* K-12 was normalized to a starting OD_600_ of 0.05 in (i) fresh MSM supplemented with 0.1% yeast—as a control, (ii) fresh MSM supplemented with 0.1% yeast and 5 mg/L MR, and (iii) Fresh MSM supplemented with 0.1% yeast and 50 mg/L MR. Triplicates of all samples were grown at 30 °C with shaking at 150 rpm until an OD_600_ of 0.15 was reached; then the cells were harvested. qRT-PCR experiments were carried out as previously explained.

Second, the difference in *azoR* gene expression between the WT *E. coli* K-12, *∆arsC*, and *∆relA* under the three conditions tested in the first experiment was investigated, and the data were analyzed by the ∆∆Ct method as detailed above.

## Results

### Systems Analysis of Transcriptomic Data

#### In Silico Analysis of *azoR* Expression Under Different Growth Conditions

Expression data for *azoR*, from 462 samples in 50 microarray datasets of different *E. coli* strains, were retrieved from the GEO (Supplementary Dataset 1) and compared in relation to different growth conditions (Supplementary Fig. S3).

**Fig. 1:**
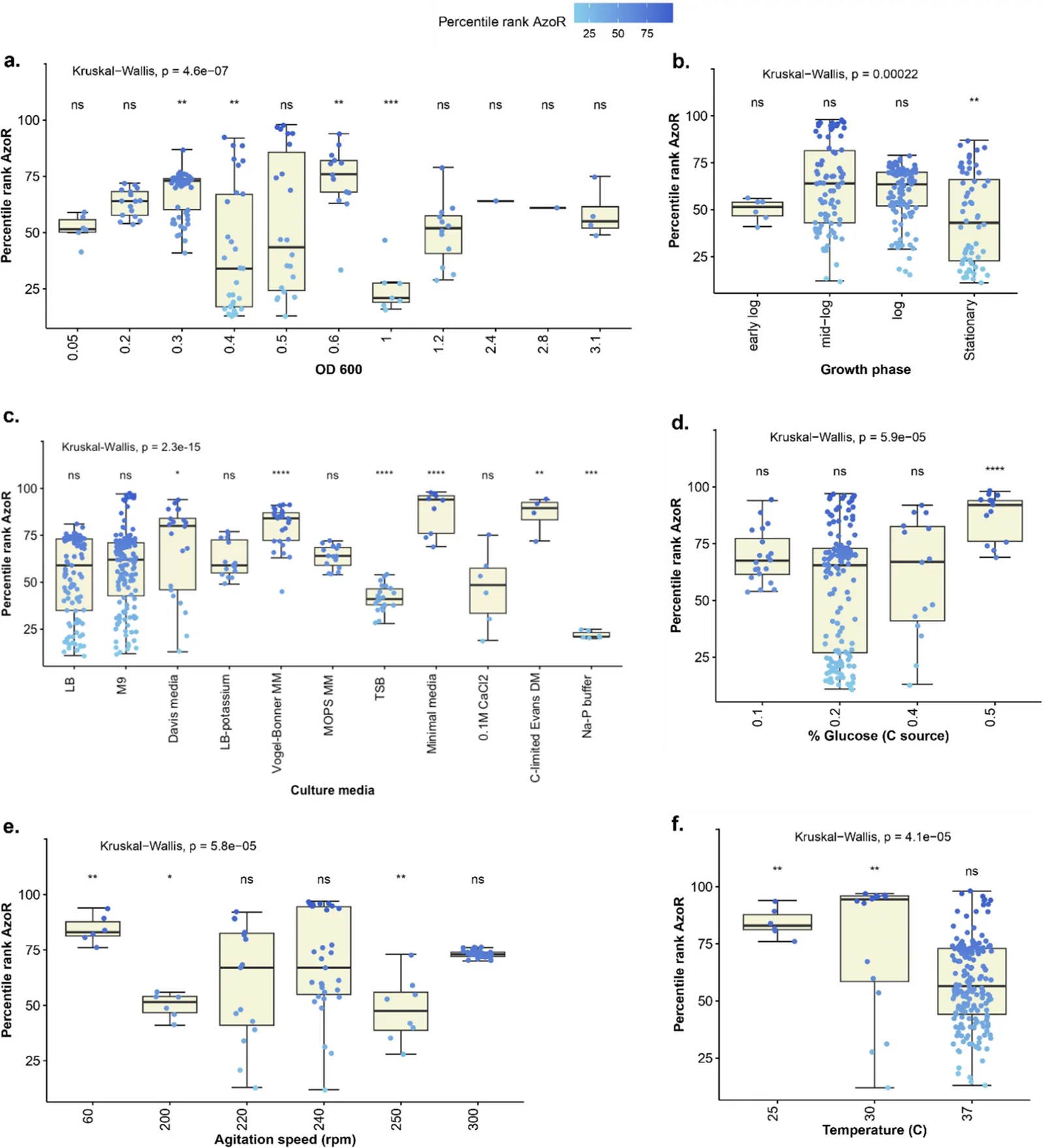
Effect of different growth conditions on the transcripts level of *azoR* in *E. coli* K-12 according to GEO expression data. Boxplots showing the computed effects of **a** culture density expressed as OD_600_, **b** growth phases, **c** culture media, **d** glucose concentrations, **e** agitation speed, and **f** incubation temperatures on *azoR *expression. The *X*-axis displays the tested conditions used in different datasets, while the *Y*-axis represents the percentile rank of the GEO expression data as statistically analyzed by R (version 4.0.2). The box spans the central 50% of the data. Significantly different categories (in comparison the median percentile rank for all values for each condition) are shown with asterisks. ns = non significant.

For the rest of the study, transcriptomic data from *E. coli* K-12 substrains (411 samples) were filtered out (Supplementary Dataset 2) and were plotted against different growth conditions to determine the conditions modulating its transcription positively or negatively. We focused on K-12 to reduce variability and because K12 was the strain used in in vitro studies. The analysis showed *azoR* to be highly expressed at the following conditions: increase in the culture optical density (OD_600_) from 0.4 to 0.7 (Fig. 1a); growth at the log phase (Fig. 1b); growth in minimal media (Fig. 1c); supplementation with high glucose concentration of 0.5% (Fig. 1d); growth at low agitation speed of 60 rpm (Fig. 1e); and at lower temperatures of 25 °C or 30 °C (Fig. 1f). On the other hand, *azoR* expression was low at OD_600_ under 0.4 and above 0.7; in stationary phase; upon growth in rich media, e.g., trypticase soy broth (TSB); at glucose concentrations of 0.1 to 0.4%; at agitation speeds of 200 to 300 rpm and at incubation temperature of 37 °C.

#### In Vitro Expression Analysis of *azoR* Under Different Growth Conditions

**Fig. 2:**
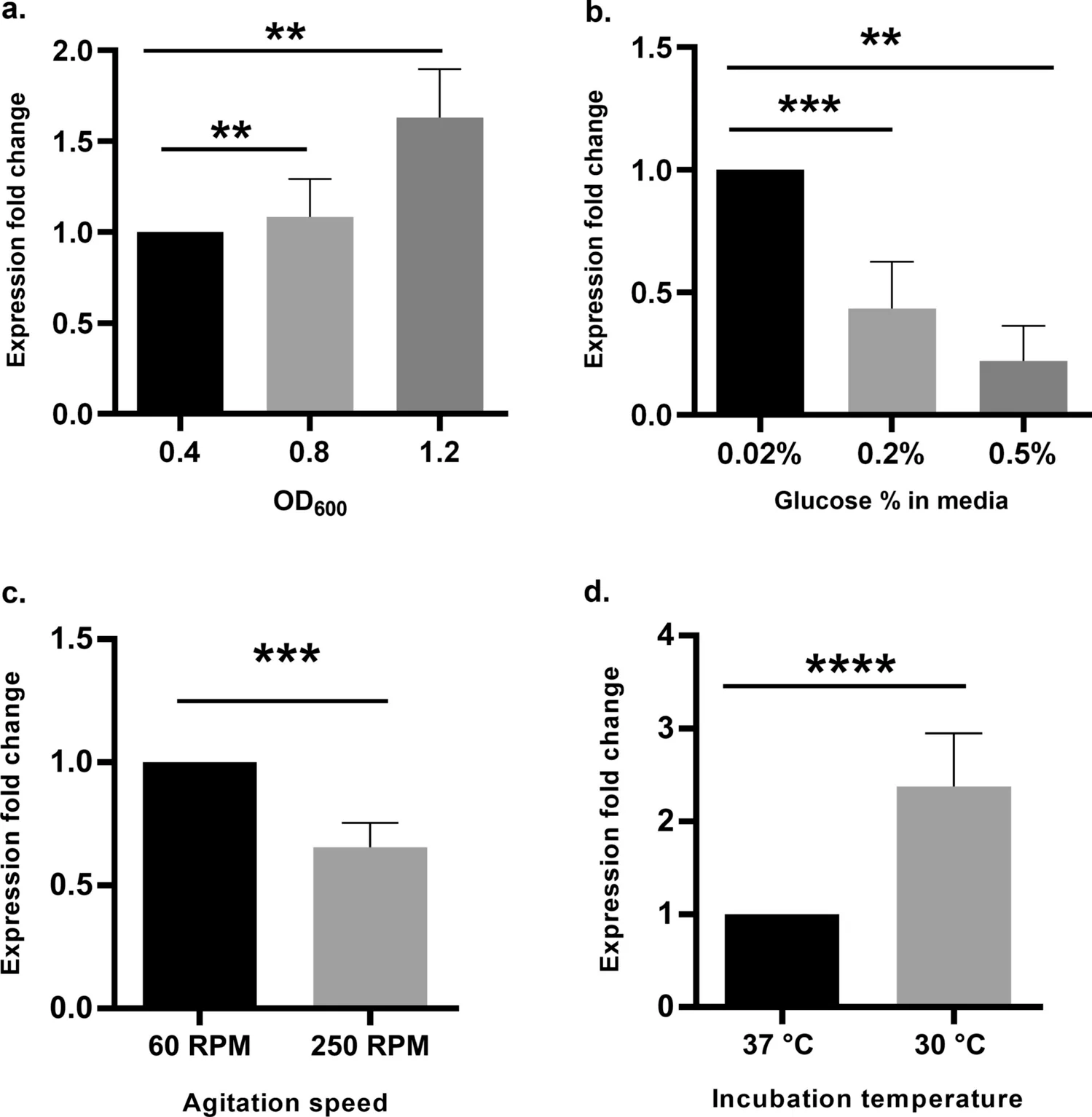
Effect of different growth conditions on *azoR* expression. Bar chart showing qRT-PCR (in vitro) results, expressed as expression fold change of *azoR*, of the effects of different **a** culture densities, **b** glucose concentrations, **c** agitation speeds, and **d** incubation temperatures on *azoR *expression. The *X*-axis represents the variables of the tested growth condition, and the *Y*-axis represents the expression fold change of *azoR*. Statistical comparisons between Ct values were tested for significance by one-way ANOVA followed by post hoc* t*-test with Tukey’s adjustment (**a** and **b**) or just *t*-tests (**c** and **d**). **p value < 0.01, ***p value < 0.001, and ****p value < 0.0001. Data presented are the mean of three replicates, and error bars represent the standard deviation

The effects of OD_600_, glucose concentration, agitation speed, and incubation temperature on the expression of *azoR* were tested by qRT-PCR to validate the predictions inferred from the computational analysis. The relative abundance of *azoR* mRNA increased as culture density increased, partially agreeing with the computational prediction since computational predictions showed a decrease in *azoR* expression over OD_600_ of 0.7 (Fig. 2a). However, *azoR* expression significantly decreased (*p* < 0.01 at 0.2% glucose and *p* < 0.001 at 0.5% glucose) at higher glucose concentrations, unlike computational predictions (Fig. 2b). On the other hand, the results of the two other tested conditions conformed to the computational predictions, as follows: a significant decrease in *azoR* expression level (*p* < 0.001) at higher agitation speed was observed (Fig. 2c). Finally, a significant increase (*p* < 0.0001) in *azoR* RNA levels was observed at 30 °C, as compared to 37 °C (Fig. 2d).

### Co-expression Profile and Analysis

#### Identification of Nine Functionally Related Co-expressed Genes with *azoR*

Data about *azoR* transcriptional “profile neighbors” were extracted from the GEO and analyzed in R software, in terms of the repetition of co-expressed genes in the different datasets, which indicates a likelihood of functional relationship to *azoR*. This analysis showed that 4345 genes have a similar expression pattern to that of the *azoR* only in a single dataset, while 1280 genes were *azoR* profile neighbors twice, 553 genes were repeated three times, 229 genes were repeated four times, 77 genes were repeated five times, 28 genes were repeated six times, five genes were repeated seven times, only one gene (*pspE*) was repeated eight times, and finally, one gene (*tesA*) was repeated ten times (Supplementary Dataset 3).

**Fig. 3:**
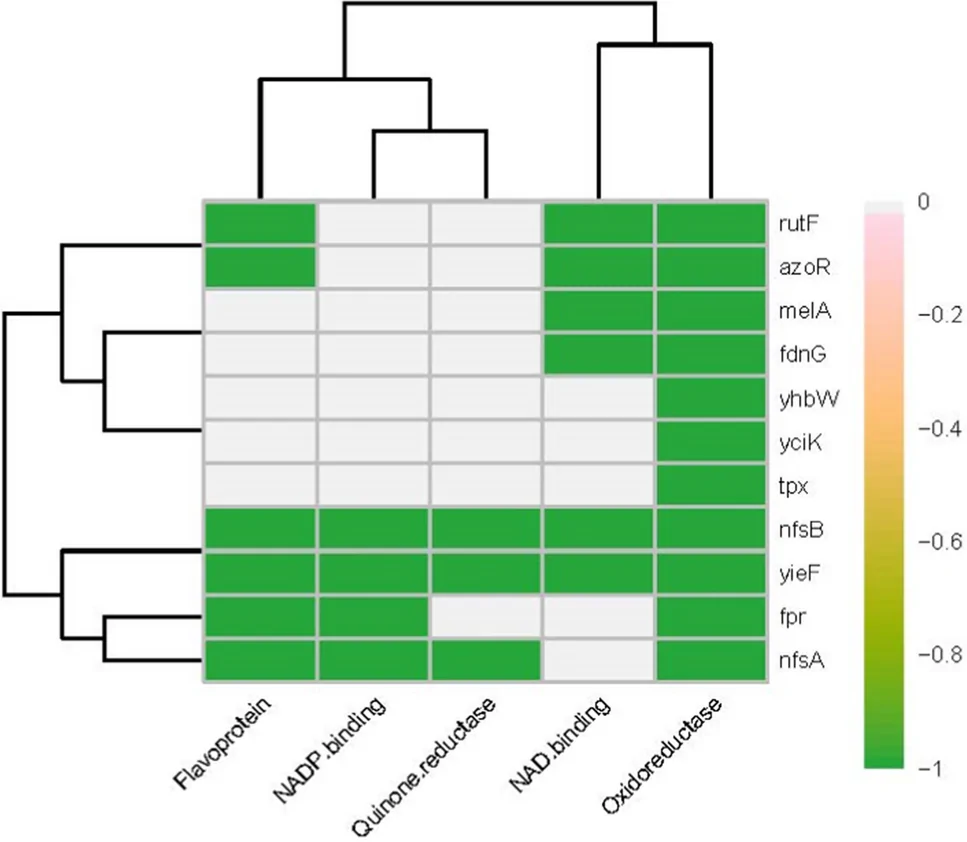
Functional clustering of *azoR *co-expressed genes. Heatmap displaying the clustering of genes co-expressed with *azoR* within at least five different GEO datasets. Clustering was generated by the DAVID software, according to the functional annotations. The horizontal axis displays the functional categories, while the vertical axis displays the genes

We used the DAVID database to identify, categorize, and classify the functional annotations of 112 genes that were profile neighbors of *azoR* at least five times (Table 1). The analysis elucidated that 34% of the genes are involved in various metabolic processes; 18% in several cellular responses, including responses to DNA damage, antibiotic resistance, stress, and others; 10% of the genes are involved in transcription regulation processes; 9% in transport, while others (with lower representation) were shown to be involved in other processes, such as DNA recombination and repair, biofilm formation, lysozyme inhibition, cell division, and proteolysis. Meanwhile, 12% of the genes remained uncharacterized. The functional clustering feature of the DAVID database was applied to the same set of genes, resulting in 13 clusters, which were filtered to eight clusters with at least two members each (Fig. S4). AzoR was clustered with ten functionally related co-expressed genes, which were annotated as oxidoreductases, flavoproteins, and reducing equivalents (FAD, FMN, NADH, or NADPH)-binders: *nfsA*, *nfsB*, *yieF*, *fdnG*, *yciK*, *fpr*, *rutF*, *yhbW*, *tpx*, and *melA* (Fig. 3).

**Table 1:** AzoR profile neighbors in at least 5 datasets and their functional annotations

Functional category	Function type	Pathway	Genes	Molecular function
Metabolic processes	Ribosome biogenesis		*rsmE*	Transferase
			*yeaK*	Hydrolase
	Biosynthetic process		*citC*	Ligase
	Purine and pyrimidine metabolism		cpdB*	Hydrolase
	Pyrimidine metabolism		*rutF*	Oxidoreductase, FMN-binding
	Purine metabolism		*add*	Hydrolase
	Xenobiotics biodegradation and metabolism		*nfsA*, *nfsB*, *yieF* (*chrR*)	Oxidoreductase, FMN-binding
	Amino acid metabolism	Valine, leucine, isoleucine metabolism	*leuC*	Lyase
			*ilvA*	
		Cysteine, methionine metabolism	*mmuM*	Transferase
		Glutathione metabolism	*chaC*	Lyase
		Glycine, serine, threonine metabolism/gluconeogenesis	*sdaA*, *ilvA*	Lyase
		Phenylalanine, tyrosine, tryptophan metabolism	*trpA*	Lyase
		D-alanine metabolism	*dadX*	
	Lipid metabolism	Sulfur metabolism, transfer RNA biogenesis	*cysQ*	Hydrolase
		Glycerophospholipid metabolism	*clsA*, gar*	Transferase
		Lipopolysaccharide metabolism	*lapA*, *lapB*	
		Lipoprotein biosynthesis	*lgt*	Transferase
		Biosynthesis of unsaturated fatty acids	*tesA*	Hydrolase
	Carbohydrate metabolism	Fructose and mannose metabolism	*fucI*, *manA*	Isomerase
			*yedP*	Hydrolase
		Glycolysis/gluconeogenesis, pentose phosphate, galactose metabolism, starch and sucrose metabolism, amino sugar and nucleotide sugar metabolism, purine metabolism	*pgm*	Isomerase, transferase
		Pentose phosphage pathway	*rbsK*	Transferase
			*rpiA*	Isomerase
		Galactose and glycerolipid metabolism	*melA*	Hydrolase, oxidoreductase
			*galT*	Transferase
	Metabolism of cofactors and vitamins	Menaquinone (secondary metabolites) biosynthesis	*menB*	Lyase
		Folate biosynthesis	*folX*	Isomerase
		Porphyrin metabolism	*hemB*, *hemD*	Lyase
	Peptidoglycan	Biosynthesis	*dacC*, *bacA*	Hydrolase
		Biogenesis/degradation	*ycjG*, dadX*	Isomerase
Cellular response		DNA damage	cpdB*, allR*, fimB*, gar*, fsr*, *yohC*	
		Antibiotic resistance	marR*, dadX*, fsr*, *marB*, *pmrD*	
		Stress	iscR*, marR*, *igaA*, *pspE* (transferase)	
		Oxidative stress	*azoR*, *fpr*	Oxidoreductase, Nucleotide-binding
			*tpx*	Oxidoreductase
		Heat	*hslU*, marR*, *loiP*	Hydrolase
		Acid stress	*hdeB*	
Transport		Amino acid	*livK*, *livJ*, *ygiS*	
		ABC transporter	*mdlA*, *mlaD* (phospholipid transport)	
		Sugar	*gntP*	
		Iron	*fecR*	Transcription regulator in response to iron starvation
		Xenobiotic transporter	*ydhC*	
		Multidrug efflux	*emrB*	
		Others	*yedA*	
Transcription regulation			*mtfA*, *yijO*, *dcuR*, *cueR*, iscR*, *phoP*, *tyrR*, *frlR*, *gar*, allR*, marR*, *bdcR*	
Putative/ uncharacterized proteins			*ybgI*, *ybjC*, *yidR*, *yjeI*, *ycjX*, *yfjQ*, *ybhg*, *yggC*, *yheU*, *yhhN*, *yafD*, *yajI*, *yjjV*, *ypfJ*	
Oxidoreductase			*fdnG*, *yhbW*	Two component system, transporter
			*yciK*	Uncharacterized oxidoreductase
Multifunctional enzyme	Regulation of nitrogen utilization, protein modification process		*glnD* (two component system)	Hydrolase
DNA replication			*holE*	Transferase
DNA recombination			*insC2*, *insC4*, *insC5*, fimB*	DNA-binding
Cell adhesion and biofilm formation			*fimI*, *yjcZ*	
Lysozyme inhibitor activity			*pliG*	Hydrolase
			*ivy*	
Cell division			*minC*	
Membrane component			*ybbJ*, *ypjD*, *yqfA*, *fxsA*	
Proteolysis			*yjfP*, *dcp*, *nlpC*, *ydgD*	Hydrolase

*Genes that have multiple functions

### Transposon Mutagenesis and Screening Identify Seven Genes Potentially Involved in *azoR* Regulation

#### Transposon Mutagenesis and Screening

A genome-wide random insertion library was constructed for *E. coli* K-12, substrain MG1655, with the Tn5 transposome, EZ-Tn5™ <KAN-2>, which confers kanamycin resistance to recipient colonies, thus allowing selection of mutants on LB agar supplemented with kanamycin. Kanamycin-resistant colonies (*n* = 4320) were picked and arrayed into 45 96-well plates.

The mutant library was screened for the ability of each clone to decolorize MR in 96-well plates. After the preliminary screening of the library, 428 mutants showed relative decolorization ≤ 65%, and 56 mutants showed relative decolorization ≥ 110%. Repetition of the test for the selected mutants, in at least two experiments with three replicates per experiment, allowed the exclusion of outliers or inconsistent results. This resulted in the selection of 15 mutants with reduced decolorization activity and five mutants with increased decolorization activity relative to the WT *E. coli*, with the same cutoffs used in the preliminary screening experiment (Table 2). The mutant decolorization percentages were statistically significantly different from that of the WT (two-tailed t-test *p* values < 0.05).

**Table 2:** Difference in decolorization ability between WT *E. coli* and library mutants, and BLAST results for the selected 20 mutants

Mutant ID	*Normalized decolorization %	BLASTn to *E. coli* K-12	Query coverage	*E*-value	Gaps	% Identity
149	43.1	Arsenate reductase (*arsC*)	100%	1e^−25^	0/64	63/64 (98%)
1533	50.3	GTP pyrophosphokinase (*relA*)	99%	0	2/551	525/551 (95%)
1454 band 1	59.5	tRNA (adenosine (37)-N6)-methyl transferase (*trmM*)	95%	0	0/534	534/534 (100%)
1454 band 2		Arsenate reductase (*arsC*)	98%	5e^−26^	0/66	65/66 (100%)
1534	46.2	tRNA (adenosine(37)-N6)-methyltransferase (*trmM*)	95%	0	0/534	534/534 (100%)
3954	53.5	tRNA (adenosine(37)-N6)-methyltransferase (*trmM*)	95%	0	2/534	532/534 (99%)
3047	42.1	tRNA (adenosine(37)-N6)-methyltransferase (*trmM*)	94%	0	1/533	530/533 (99%)
3968 band 1	58.8	Glycerol-3-phosphate acyltransferase (*plsY*)	100%	0	3/497	493/497 (99%)
3968 band 2		tRNA (adenosine(37)-N6)-methyltransferase (*trmM*)	93%	0	0/534	534/534 (100%)
3997	53.3	tRNA (adenosine(37)-N6)-methyltransferase (*trmM*)	95%	0	0/534	534/534 (100%)
1922	49.8	tRNA (adenosine(37)-N6)-methyltransferase (*trmM*)	100%	0	1/511	487/511 (95%)
3988 band 1	38.04	Arsenate reductase (*arsC*)	72%	3e^−28^	0/66	66/66 (100%)
3988 band 2		Glutamate cysteine ligase (*gshA*)	98%	0	1/784	764/784 (97%)
3988 band 3		Glutamate cysteine ligase (*gshA*)	100%	0	3/478	459/478 (96%)
1015 band 1	64.76	Arsenate reductase (*arsC*)	72%	2e^−26^	0/66	65/66 (98%)
1015 band 2		No similarity found				
1240	64.04	Glutamine synthetase adenylyltransferase (*glnE*)	100%	0	0/455	442/455 (97%)
2995	61.7	Arsenate reductase (*arsC*)	70%	3e^−28^	0/66	66/66 (100%)
1942 band 1	65	No similarity found				
1942 band 2		No similarity found				
3018	32.3	No similarity found				
266 band 1	127.1	No similarity found				
266 band 2		YqcC family protein	100%	1e-04	0/21	21/21 (100%)
2474	122.3	tRNA (adenosine(37)-N6)-methyltransferase (*trmM*)	95%	0	1/533	532/533 (99%)
269	118.3	tRNA (adenosine(37)-N6)-methyltransferase (*trmM*)	15%	1e^−72^	1/180	170/180 (94%)
217	114.6	No similarity found				
268 band 1	110.7	No similarity found				
268 band 2		No similarity found				

*Decolorization data are the mean of three replicates. Decolorization % are statistically different from that of the WT *E. coli*, *p* value < 0.05

The transposon insertion sites for the chosen strains were determined by RATE PCR, followed by sequencing of PCR products. Seven isolates showed more than one band, and in those cases, DNA from all bands was extracted. Sequences with poor quality or with no hit to the *E. coli* genome were discarded, while good-quality sequences were trimmed then aligned against the *E. coli* K-12 substrain MG1655 genome, and the first hit in each case was retrieved **(**Table 2**)**.

#### Confirmation of the Involvement of *arsC* and *relA* in the Decolorization Process

**Fig. 4:**
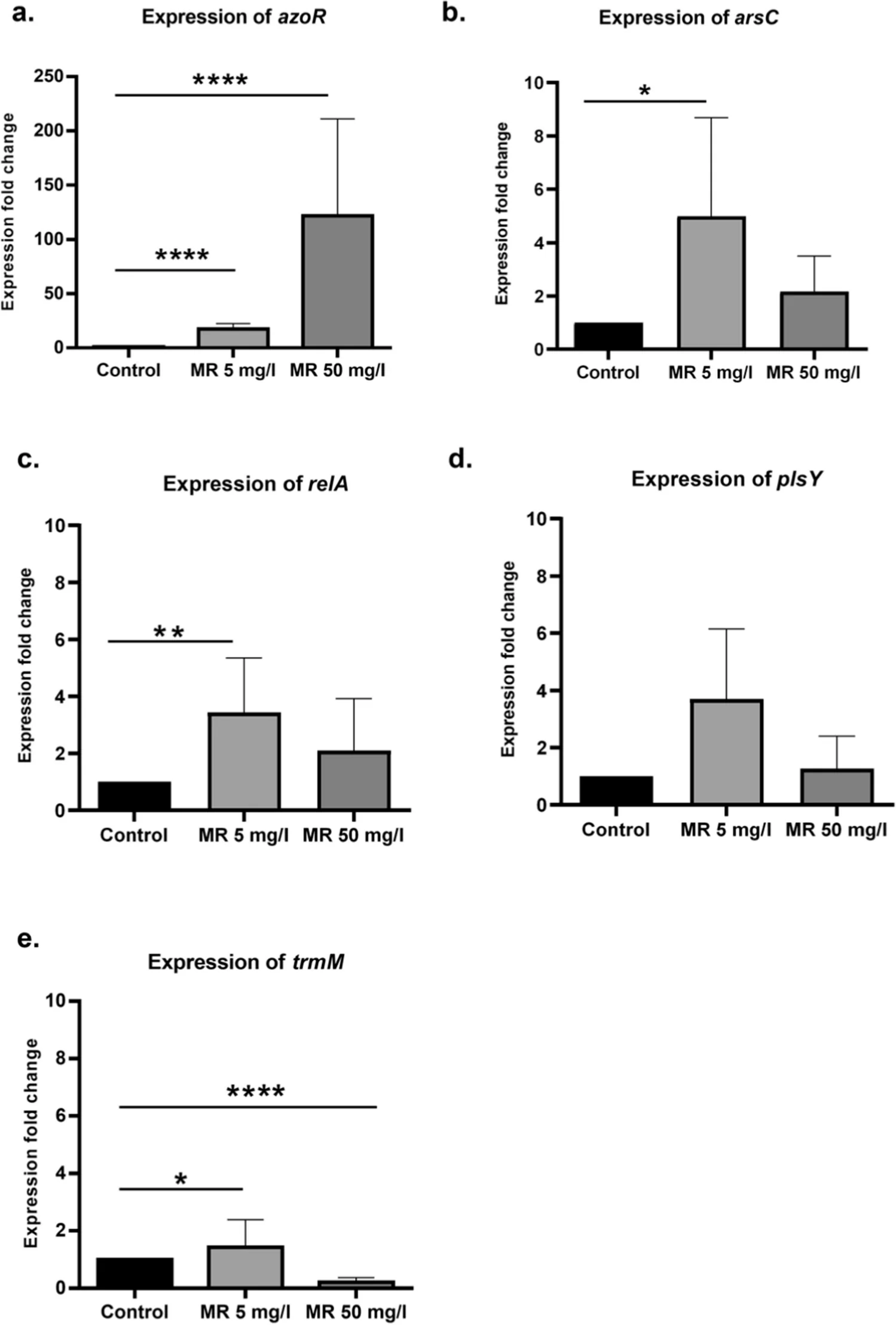
Effect of different MR concentrations on the expression of candidate genes. Bar charts showing the differences in gene expression of **a** *azoR*, **b** *arsC*, **c** *relA*, **d** *plsY*, and **e** *trmM* upon exposure to different MR concentrations. The *X*-axis represents the samples exposed to different MR concentrations, and the *Y*-axis represents the expression fold change of the gene of interest. Comparisons between Ct values were tested for statistical significance by one-way ANOVA followed by post hoc* t*-test with Tukey’s adjustment. *p value < 0.05, **p value < 0.01, ***p value < 0.001, and ****p value < 0.0001. The data are the mean of three replicates, and error bars represent the standard deviation

The expression levels of the candidate regulatory genes of interest (*arsC*, *relA*, *plsY*, and *trmM*) versus *azoR* in the WT *E. coli* were determined after the bacteria were exposed to MR concentrations of 5 and 50 mg/L. These levels were compared to their expression levels in the absence of the dye. *azoR*, *arsC*, *relA*, and *trmM* were differentially expressed at one or both MR concentrations. The expression of *azoR* significantly increased (*p* < 0.0001) in presence of both MR concentrations (Fig. 4a), whereas *arsC* and *relA* expression significantly increased (*p* < 0.05 and *p* < 0.01, respectively) only in response to 5 mg/L dye, despite their genetic upregulation at both concentrations (Fig. 4b and c). Expression of *plsY* did not significantly increase in response to 5 mg/L dye (Fig. 4d). Finally, the expression of *trmM* increased (*p* < 0.05) only in response to 5 mg/L dye (Fig. 4e).

*∆arsC* and *∆relA* were further studied based on their promising involvement in the decolorization process and possible feedback or compensatory activity on *azoR*, as elucidated in the previous phenotypic and genotypic experiments. In the second RT-qPCR assay, the gene expression levels of *azoR* in the WT *E. coli* and in both *arsC* and *relA* mutants were compared upon stimulation with MR concentrations of 5 and 50 mg/L, with the expression level of *azoR* in WT *E. coli* (unexposed to the dye) used as control.

**Fig. 5:**
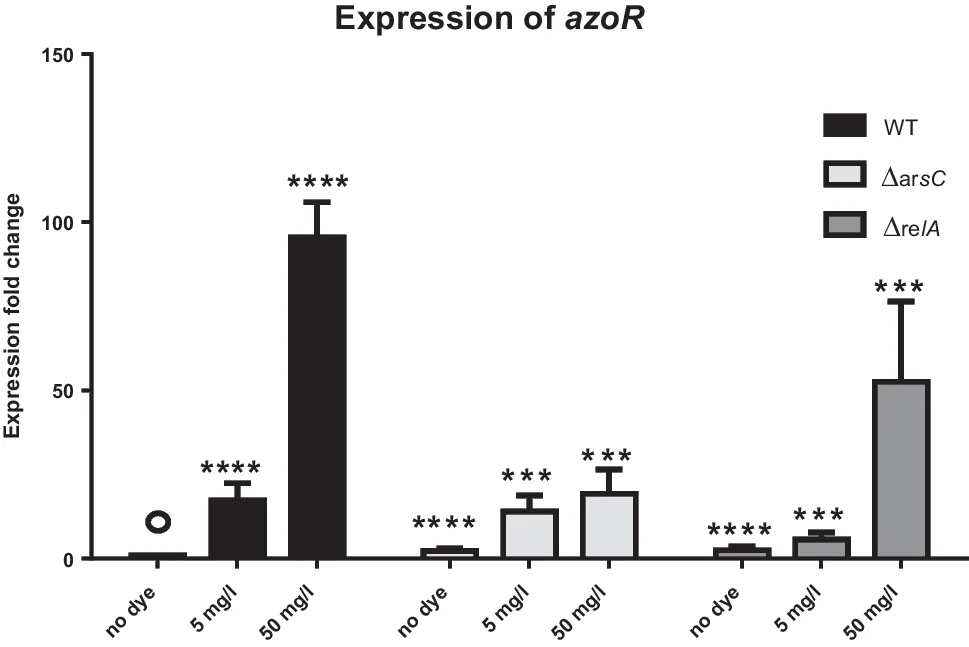
Difference in *azoR *expression in WT* E. coli*, and mutants of interest. Bar charts showing the difference in *azoR* expression levels between WT* E. coli*, *∆arsC*, and *∆relA* upon stimulation with 5 mg/L and 50 mg/L MR as compared to control experiment with no dye added. The *X*-axis represents the different strains exposed to different MR concentrations. Each strain was exposed to three MR concentrations (0, 5, and 50 mg/L). The *Y*-axis represents the expression fold change for the gene of interest. Comparisons between Ct values were tested for statistical significance by one-way ANOVA followed by post hoc* t*-test with Tukey’s adjustment for differences from the “no-dye” WT control (indicated by °). ***p value < 0.001 and ****p value < 0.0001. The data are the mean of three replicates and error bars represent the standard deviation

The transcriptional activity of *azoR* in both *∆arsC* and *∆relA*, compared to that in the WT *E. coli*, significantly decreased (*p* < 0.001) upon induction by MR. This significant decrease may explain the decreased phenotypic activity of these mutants (Fig. 5).

## Discussion

In this study, we investigated the regulation of *azoR* expression in *E. coli* K-12 to better understand the physiological role these enzymes might play. This role is particularly interesting to fully uncover because, although azo dyes are mostly connected to human activity, the presence of bacterial azoreductases that predate humans suggests that these enzymes may have different physiological roles in bacteria [9].

Combining a systems approach, involving computational transcriptome analysis, with a reductionist qRT- PCR-based approach demonstrated the following:

First, *azoR *expression was higher at 30 °C than at 37 °C, suggesting that 30 °C is the optimal temperature for decolorization activity and is consistent with previous results that showed an enhanced in vitro activity of azoreductases at 30 °C rather than 37 °C [21].

Second, increasing agitation was predicted to decrease *azoR *expression, conforming  with previous phenotypic results [22]. This could be a result of increased oxygen transfer, which decreases enzymatic activities [23], or competition between oxygen and *azoR* over electron donors [24].

Third, increasing culture density from 0.4 to 0.8 to 1.2 was found to consistently increase *azoR* expression, partially conforming to computational analyses which predicted that initially increasing OD increases *azoR* expression to a certain extent after which the activity decreases, which is consistent with a previous study [25].

Finally, increasing glucose concentration decreased *azoR* transcription, unlike the results of computational analysis, in which *azoR* expression values tended to increase with increasing glucose concentration; however, the GEO-retrieved data were quite heterogeneous. For example, for glucose concentration of 0.2%, there were two subpopulations of *azoR* expression values (Supplementary Fig. S5).

The experimental effect of glucose concentration (Fig. 2b), which was observed under controlled conditions (MSM minimal media and OD = 0.5), could be attributed to the abiotic stress the bacteria encounter at a decreased glucose concentration while surviving in a carbon-depleted minimal media as opposed to higher glucose concentrations. Using glucose as a carbon source was found to phenotypically enhance azo-reducing activity in several studies, until a certain threshold after which the decolorization activity decreases, probably due to preferential consumption of the dye rather than the external carbon source [26, 27].

**Fig. 6:**
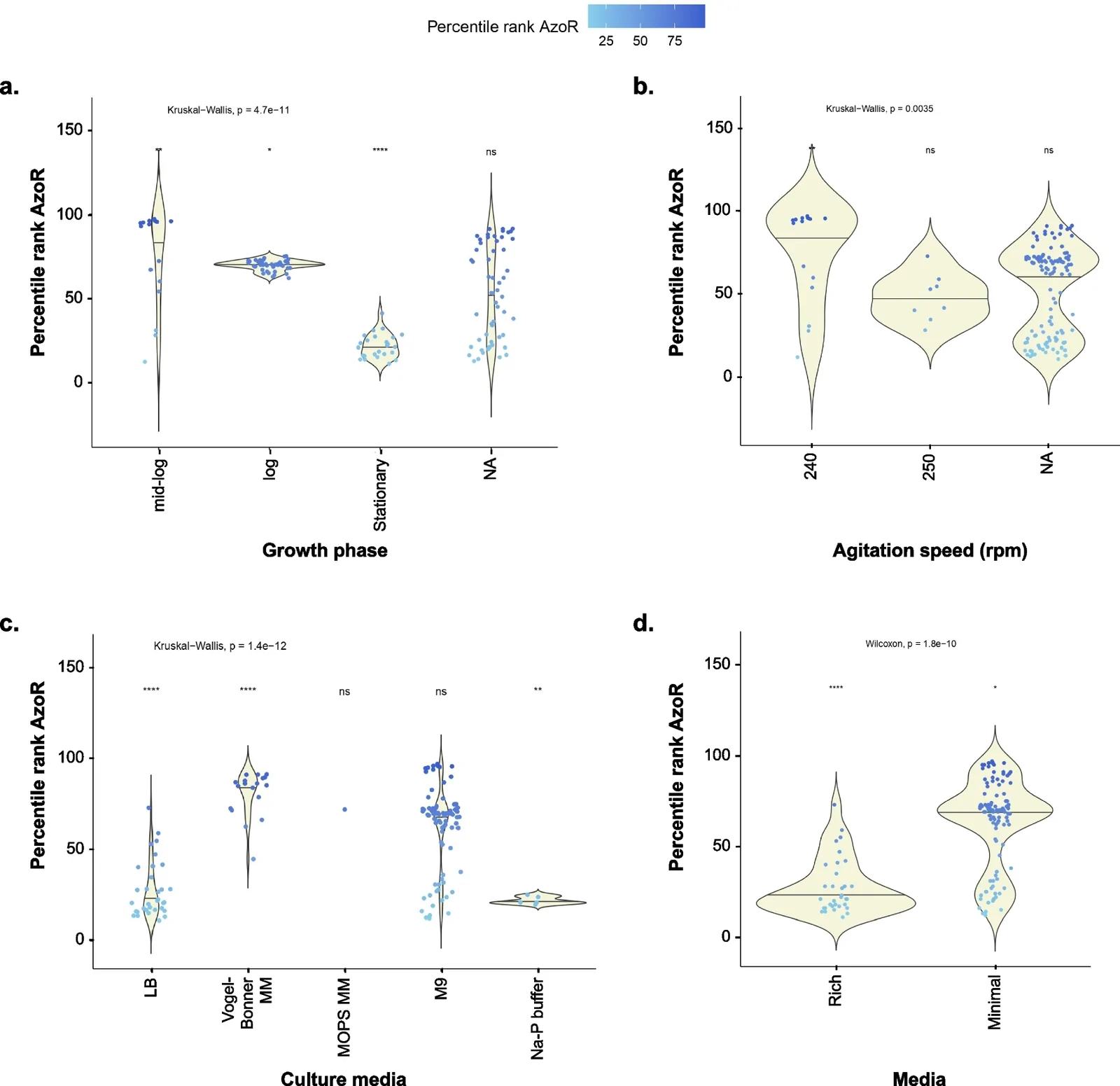
Variations of *azoR* expression levels in *E. coli* K-12 within the 142 samples that had been grown in 0.2% glucose, according to GEO expression data. Significant variations of **a** growth phase, **b** agitation speed, **c** culture media, and **d** type of media within. Significantly different categories (in comparison the median percentile rank for all values for each condition) are shown with asterisks. ns = non significant.

The observed discrepancy between in vitro experiments and in silico analysis of some factors, especially glucose %, is most likely due to the controlled in vitro experimental setting—unlike the heterogeneity of GEO datasets. For example, the culture density effect was consistent in vitro (Fig. 2a), while the computational analysis showed a large spread in expression values at OD 0.4 and 0.5 (Fig. 1a). This is because the in vitro experiment was conducted in one culture medium, at a fixed agitation speed (250 rpm) and fixed culture conditions, while the computational analysis was conducted on data from all available conditions (Fig. 1 and Supplementary Fig. S3). Likewise, the discrepancy in measured *azoR* expression under different glucose % might be due to the wide variation between other confounding experimental conditions. For example, out of 190 GEO experiments for which glucose concentration was stated, 142 experiments (74.7%) were conducted at a glucose concentration of 0.2%, but a wide variation of agitation speed, OD, and type of media, and these variations had significant effects on *azoR* expression (Fig. 6).

Using the GEO datasets for co-expression analysis to determine genes with similar expression patterns to that of *azoR*, followed by functional categorization, allowed us to functionally cluster *azoR* with ten co-expressed genes, all of them encoding oxidoreductases, namely *melA*, *tpx*, *yhbW*, *yciK*, *fdnG*, *fpr*, *nfsA*, *nfsB*, *rutF*, and *chrR* (*yieF*). Three of the clustered genes were identified as quinone reductase genes: *nfsA*, *nfsB*, and *yieF*. They are also clustered based on the required cofactor as energy donor for the oxidoreduction reaction. In this context, *rutF*, *azoR*, *melA*, and *fdnG* encode NAD-binding enzymes, *nfsA* and *fpr* encode NADP-binding enzymes, while *nfsB* and *yieF* encode both NAD- and NADP-binding enzymes. Finally, *rutF*, *azoR*, *nfsA*, *nfsB*, *fpr*, and *yieF* encode flavoprotein (FMN/FAD)-binding enzymes. Details about those genes, encoded functions, and their interrelations are provided in Supplementary Material.

As previously mentioned, azoreduction is an electron transport process that utilizes electron donors for subsequent reduction of azo dyes [28]. Among the components involved in the electron transport chain (ETC) are dehydrogenases, such as the formate dehydrogenase N alpha subunit (FdnG), which is able to transfer electrons to terminal reductases using quinones as redox mediators [29]. This co-occurrence in the ETC could explain the correlation between *fdnG* and *azoR* with probable involvement in the azo reduction process, and agrees with the upregulation of another dehydrogenase gene, *fdhF*, up to 4.5 folds upon exposure to AR18, and its subsequent downregulation upon enzyme deletion [13]. Likewise, *yciK*, which encodes a putative yet unannotated oxidoreductase, was among *azoR* profile neighbors in five datasets in our study, and was also upregulated in response to the azo dye AR18 [13]. This suggests its probable role as an azoreductase, a hypothesis that requires further investigation.

In addition to computational systems analysis, another system approach to comprehensively study *azoR* regulation was to construct a Tn5 transposon insertion mutant library of 4320 kanamycin-resistant mutants in *E. coli* K-12 MG1655. When screened for MR decolorization ability, 20 mutants were found to have significantly different decolorizing abilities from the WT strain, suggesting that the transposon interrupted key genes in the reduction process. Transposon insertions were identified in seven different genes, namely *arsC*, *relA*, *plsY*, *trmM*, *gshA*, *glnE*, and the gene encoding the hypothetical “YqcC family protein.”

Arsenate reductase (*arsC*) is a part of the arsenic resistance operon (*ars*) in *E. coli*, which provides resistance against As^5+^ [30–32]. Other *ars* operon products include the protein ArsH, which is an FMN reductase with a demonstrated ability to reduce chromate [33], ferric iron [33, 34], quinones [35] and, interestingly, azo dyes [36–38] as well as a quencher of oxidative stress [39]. This is particularly interesting because, although the *E. coli* K-12 genome lacks an *arsH* gene [39] in its *ars* operon, the redox *arsC* gene product showed in our study a similar azo dye-reducing ability in both phenotypic and genetic assays. We therefore suggest that the arsenic resistance operon is also involved in MR decolorization and acts as a stress quencher brought about by exposure to azo dyes.

The second transposon insertion was detected in the *relA* gene, encoding a GDP/GTP pyrophosphokinase. RelA is a stringent factor that plays an important role in bacterial adaptation to amino acid starvation through the production of guanosine tetraphosphate (ppGpp) and guanosine pentaphosphate (pppGpp) [40]. Besides, glutamate excretion as a result of amino acid starvation is a *relA*-dependent process [41]. Another pathway for the stimulation of *relA* transcription is a result of nitrogen starvation or glutamine limitation by the σ^54^-dependent response regulator NtrC through the GlnD pathway [42]. Therefore, an insertion in the *relA* gene is expected to cause drainage of the glutamine pool as well as accumulation of glutamate, under nitrogen and amino acid starvation, respectively.

To the best of our knowledge, no prior studies correlated the products of *relA* and *azoR*. Here, the threefold upregulation of *relA* in response to MR exposure, and subsequent decrease in *azoR* activity and transcription upon insertion in *relA*, suggest they are correlated. We propose this upregulation as a stringent response by which the cells adapt to their exposure to azo dyes.

An insertion was identified in the putative glycerol-3-phosphate acyltransferase gene (*plsY* or *ygiH*) which plays a role in phospholipid biosynthesis [43]. *plsY* showed a non-significant upregulation in response to MR exposure, and its mutant showed a defected azoreductase activity in vitro. Surprisingly, this coincides with the upregulation of another acyl-glyerol-3-phosphate acyltransferase in *Staphylococcus aureus* upon exposure to Sudan III [44].

Seven transposon insertions were identified in tRNA (adenosine(37)-N6)-methyltransferase (*trmM* or *yfiC*). This gene’s product methylates adenosine 37 in tRNA_1_^Val^ at the N6 position, which helps stabilize the tRNA structure. YfiC was also found to resist osmotic and oxidative stress [45].

Another insertion was identified in the glutamine synthetase adenylyl transferase gene (*glnE*). Glutamine synthetase (GS) catalyzes glutamine synthesis which, along with glutamate and other metabolites, is required for nitrogen assimilation. GlnE activates and reduces GS activity under low and high nitrogen levels, respectively. A reduction in GS activity is regulated by cellular glutamine concentration [46], and therefore an insertion in the *glnE* gene might result in a depletion of glutamate levels due to the continuous GS activity. This hypothesis is supported by a reported transcriptional upregulation of *glnR*, the glutamine synthetase repressor in *S. aureus*, upon exposure to the azo dye, Sudan III [44].

An insertion was observed in gamma-glutamyl cysteine synthase (*gshA*), which is involved in glutathione (GSH) biosynthesis, a thiol that plays a crucial role in protection against stress, as an antioxidant and a redox buffer [47]. An insertion in *gshA* had no effect on *E. coli*’s resistance to oxidative damage and gamma radiation; however, it contributed to thiol-specific stress [48]. Moreover, the lack of GSH, as a result of *gshA* inactivation, increased the sensitivity of *E. coli* to both mercury and arsenite [49]. *azoR* expression increased in *E. coli* in response to quinones to prevent thiol-specific stress, which might have led to GSH depletion [9], but the effect of GSH absence on *azoR* transcripts in *E. coli* was not studied further. We, therefore, propose that the decrease in azo reduction in this clone resulted from the absence of glutathione and therefore increased oxidative stress. In other terms, this mutation presumably leads to the accumulation of glutamate.

The phenotypic decrease in azo reduction upon insertions in *gshA* or *glnE* suggests that *azoR* might be involved in ammonium assimilation. Our computational analysis supports this suggestion as it indicated the co-expression between *azoR* and *glnD* in five different datasets. GlnD also plays an important role in nitrogen assimilation regulation and metabolism [50].

Finally, an ambiguous mutation in a 21-bp segment of the gene encoding the hypothetical protein YqcC was identified in a phenotype that showed higher azo-reducing activity than the WT *E. coli*. The exact function of the protein is unknown; nonetheless, it was reported to have a biofilm-related function [51].

To validate some of the above predictions, we measured the transcripts of *arsC*, *relA*, *plsY*, and *trmM*, as well as that of the well-known *azoR* gene in WT *E. coli* K-12 upon exposure to two different MR concentrations. While the transcription of the four genes increased in response to the lower MR concentration, only *arsC* and *relA* transcriptionally increased at both MR concentrations. This suggests a regulatory or feedback mechanism, resulting in a compensatory action through the genetic upregulation of these genes—probably in response to the oxidative stress imposed on the bacterial cells by exposure to the dye. The significant decrease in *azoR* transcriptional activity in *∆arsC* and *∆relA* mutants suggests a role these two genes might play in regulating azoreductase activity and justifies the observed phenotypic decrease in decolorization activity by both strains. It also suggests a potential relation between *arsC* and *arsH*, as both exhibit similar azo dye-reducing activity and play a part in the arsenical resistance operon.

## Conclusion and Outlook

In conclusion, this work followed a systems approach to study *azoR* gene expression in *E. coli* K-12 combining computational and experimental genome-wide mutagenesis tools. Based on the computationally analyzed transcriptomic data, we hypothesize the possible regulation of *azoR* by either the SoxRS or MarRAB regulons or both; however, this hypothesis needs further investigation. Our work also suggests that *yciK* is a probable azoreductase and that the arsenic resistance operon is involved in azo dye decolorization.

Using a loss-of-function approach by transposon mutagenesis coupled with qRT-PCR, we identified *arsC* and *relA* as probable *azoR* regulators based on the significant transcriptional upregulation in *E. coli* K-12 upon exposure to MR. This hypothesis is backed by the consequent decrease in *azoR* transcriptional activity in *∆arsC* and *∆relA* strains as compared to the WT *E. coli*.

This work highlights the importance of better understanding the physiological role azoreductases might play. Future work includes precisely deleting these genes and defining their phenotypes along with subsequent complementation assays.

## Electronic Supplementary Material

Below is the link to the electronic supplementary material

Supplementary Material 1

Supplementary Material 2

## Data Availability

No datasets were generated or analyzed during the current study.
