# Supplementary material for: Genome-Scale Investigation of the Regulation of azoR Expression in Escherichia coli Using Computational Analysis and Transposon Mutagenesis
Source: Microb Ecol. 2024 May 1;87(1):63. doi: 10.1007/s00248-024-02380-5 (PMC11062982; doi:10.1007/s00248-024-02380-5)
Supplement: Supplementary file 2 — Supplementary Material 2 [file 248_2024_2380_MOESM2_ESM.pdf]

Genome-scale investigation of the regulation of *azoR* expression in *Escherichia coli* using computational analysis and transposon mutagenesis

Mona A. Salem[1], Hanzada T. Nour El-Din[2], Abdelgawad M. Hashem[1], Ramy K. Aziz[2,3,4*]

[1]Department of Microbiology, Faculty of Pharmacy, The British University in Egypt (BUE), El-Sherouk City 11837, Egypt.
[2]Department of Microbiology and Immunology, Faculty of Pharmacy, Cairo University, Qasr El-Ainy St., Cairo 11562, Egypt.
[3]Center for Genome and Microbiome Research, Cairo University, Cairo 11562, Egypt.
[4]Microbiology and Immunology Research Program, Children's Cancer Hospital Egypt 57357, Cairo 11617, Egypt.

Short title: Regulation of *azoR* expression in *E. coli*

* To whom correspondence should be addressed. E-mail: ramy.aziz@pharma.cu.edu.eg

SUPPLEMENTARY MATERIAL

__Content:__

- Supplementary Methods
- Supplementary Discussion
- **Table S1:** List of primers used in the study
- **Figure S1:** MR calibration curves
- **Figure S2:** Differences in decolorization of MR by the WT strain of *E. coli* K-12 and Δ*azoR*
- **Figure S3:** Computationally predicted effects of six growth conditions on the expression of *azoR* in *E. coli* strains
- **Figure S4:** Functional clustering of *azoR* co-expressed genes in at least five GEO datasets
- **Figure S5:** Bimodal distribution of GEO-retrieved azoR expression data at glucose 0.2% concentrations.

Supplementary Methods:

Optimization of screening assay for azoreductase activity

Tests for method optimization were carried out to determine the most efficient wavelength for decolorization measurement, as well as the optimal incubation time and culture media. The test was carried out in sterile, flat bottomed 96-well plates following the method developed by Lucas et al. [1] with some modifications. A random plate from the library was chosen for the assay. The WT *E. coli* was also added.

A. Determination of the most efficient wavelength for decolorization measurement

Overnight cultures of both the mutants and the WT were added to the assay plate in a 1:10 dilution in a final volume of 150 µl MSM supplemented with 0.1% yeast and 50 mg/l MR. Plates were incubated at 30 °C with shaking at 150 rpm for 24 h. A 96-well plate reader, equipped with four monochromatic filters (405, 450, 490 and 630 nm), was used for decolorization measurement. The absorbance of the prepared plates was measured at the four above mentioned wavelengths before and after 24 h. incubation to choose the best wavelength for the assay. The decolorization ability of both the WT *E. coli* and the mutants, expressed as a percentage, was calculated using the following rule:

$$\text{Decolorization percentage (\%)}=\frac{\text{Initial absorbance-Final abosrbance}}{\text{Initial absorbance}}\times 100$$

To correct for the bacterial presence, the absorbance of each well at 630 nm was subtracted from its absorbance at each specified wavelength after 24 hrs.

For the WT, the decolorization percentages at the different wavelengths were: 66.9%, 71.9%, 49.86%, and -120.1%, respectively. Since the highest decolorization activity was obtained at 450 nm, all subsequent screening was carried out at this wavelength.

Another way for validating the optimum wavelength was through determining the λ_{max} for MR by preparing different MR concentrations and measuring their absorbance at all available wavelength filters using the plate reader as well as using the UV spectrophotometer. The highest absorbance was shown at 450 nm using the plate reader and at 430 nm using the UV spectrophotometer. Standard curves were constructed to ensure linearity using Microsoft Excel (Version 16.0.4266.1003) **(**

Fig. S1).

B. Determination of the optimal incubation time and culture media for the assay

The WT *E. coli* and Δ*azoR* strain were used for optimization of the best incubation time and culture media for the assay. The two strains were selected for assay optimization of maximum decolorization differences between the WT and the tested mutants.

The bacterial cells were cultured in two culture media: (i) MSM supplemented with 0.1% yeast, with kanamycin (for Δ*azoR*) and without kanamycin (for WT) and (ii) LB broth supplemented with kanamycin (for Δ*azoR*) and without kanamycin (for WT). This was followed by an overnight incubation at 37 °C and with shaking at 150 rpm. The following day, the bacterial OD was measured at a wavelength of 630 nm using a 96-well plate reader and then diluted 1:10 in fresh media. The assay plate was filled as previously described and the plates were incubated at 30 °C with shaking at

150 rpm. The absorbance was measured, at both 630 nm and 450 nm, at a 2-hour time interval for 24 hours.

Δ*azoR* was eventually able to decolorize MR at a limited and delayed pattern as compared to the WT when cultured in both LB and MSM (**Fig. S2**). However, unlike with enriched LB medium, carrying out the assay in MSM maximized the differences in decolorization activity between both the WT and Δ*azoR* strains. Hence, the screening assay was later carried out in MSM, and the decolorization activity was measured both at 0 time and after 16 h. incubation.

Supplementary Discussion:

Details about coexpressed genes:

nfsA and *nfsB* were co-expressed with *azoR* in six and five datasets, respectively. Both genes express nitroreductases whose function is similar to *azoR* in using an electron transfer mechanism [2]. Although both enzymes were previously reported to reduce tartrazine, albeit in a limited way [3], they were unable to reduce MR [2]. Intriguingly, *nfsA* is co-transcribed with *ybjC*, which was another *azoR* profile neighbor in five datasets [4]. Moreover, the expression of both *nfsA* and *ybjC* is coregulated by the xenobiotic paraquat via the SoxRS system, which regulates the cellular response to oxidative stress. *nfsA* and *ybjC* are also members of another regulon, MarRAB, which regulates the response to antibiotics [4]. Interestingly, MarA was shown to control the expression of a number of genes, including *fpr*, *yhbW*, *hemB*, *marB*, *marR*, *tpx*, and *ybjC*, all of which showed a co-expressed profile to *azoR* in several datasets [5]. Further studies are required to investigate the possibility of *azoR* regulation by any of these regulons.

yieF, which is co-expressed with *azoR* in five datasets, encodes a NAD(P)H-dependent oxidoreductase capable of reducing chromate, uranyl and prodrugs. YieF acts as a quinone reductase, thus providing resistance to oxidative stress [6, 7], and YieF homolog in *P. aeruginosa* was described to reduce azo dyes [8].

fpr, which encodes an electron transporter, was co-expressed with *azoR* in five datasets. This gene product transports electrons between ferredoxin/flavodoxin and NAD(P)H [9].

RutF is the last reductase whose encoding gene was clustered with *azoR*. It is a flavin reductase that plays a role in pyrimidine degradation together with other *rut* operon [10]. The *rut* operon is regulated by the RutR regulator [11] and NtrC, which also activates glutamine synthetase (*glnA*) and activates the transcription of the *relA* gene under nitrogen starvation conditions [12].

tpx encodes a thiol hydroperoxide peroxidase and a major antioxidant under anaerobic conditions. It utilizes reducing equivalents from thioredoxin to reduce hydroperoxidase. It also protects the cells against oxidative stress [13].

melA encodes an alpha galactosidase involved in galactose metabolism when bacterial cells utilize melibiose as a sole carbon source. Its correlation with *azoR* could be attributed to its ability to bind NAD^+ for better enzymatic stabilization [14].

Table S1: List of primers used in the study

Primer	Primer sequence (5`-3`)	Description	Source
ECoK12-azoR-FP	GCGGCACCGATGTATAACTT	Used for qRT-PCR experiments	Designed
ECoK12-azoR-RP	GTCCGTTGGTCCATCTTTGT	Used for qRT-PCR experiments	Designed
Tn5_Kan_Set1-FP	CAATCAGGTGCGACAATCTA	Used for confirmation of transposon insertion	Designed
Tn5_Kan_Set1-RP	CCATGAGTGACGACTGAATC	Used for confirmation of transposon insertion	Designed
Inv-1	ATGGCTCATAACACCCCTTGTATTA	Used for rapid amplification of transposon mutants	[15]
KAN2-RP1	GCAATGTAACATCAGAGATTTTGAG	Used for sequencing transposon mutants	Supplied in the EZ-Tn5™ <KAN-2>Tnp Transposome kit
ECoK12-arsC-FP	TGAGCAACATTACCATTTATCACA	Used for qRT-PCR experiments	Designed
ECoK12-arsC-RP	GTTGGCGGAGTTTCCAGATA	Used for qRT-PCR experiments	Designed
ECoK12-trmM-FP	AGTGCTCGACACCTCAACG	Used for qRT-PCR experiments	Designed
ECoK12-trmM-RP	GCTTAATGCCAGCTCCGTAA	Used for qRT-PCR experiments	Designed
ECoK12-relA-FP	AAATCCTTGACGACGAGCTG	Used for qRT-PCR experiments	Designed
ECoK12-relA-RP	TGCAGGAAGTTCACCATCTG	Used for qRT-PCR experiments	Designed
ECoK12-plsY-FP	CTGGGATCTCACCGGAGTAA	Used for qRT-PCR experiments	Designed
ECoK12-plsY-RP	GGATCAGGCAAGAGAGCATC	Used for qRT-PCR experiments	Designed
ECoK12-ihfB-FP	GATAGAAAGACTTGCCACCCA	Housekeeping gene used for qRT-PCR experiments	[16]
ECoK12-ihfB-RP	CCAGTTCTACTTTATCGCCAG	Housekeeping gene used for qRT-PCR experiments	[16]

Figure S1

a)

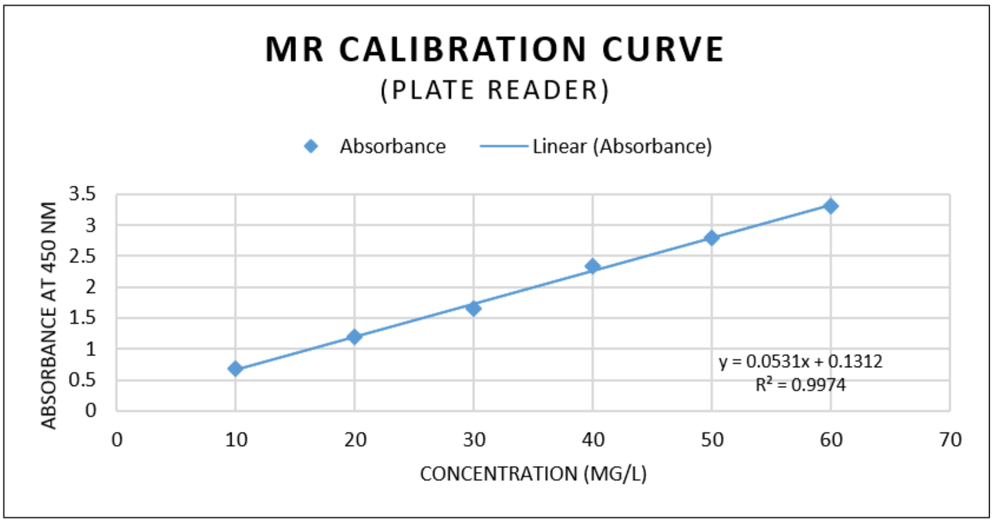

b)

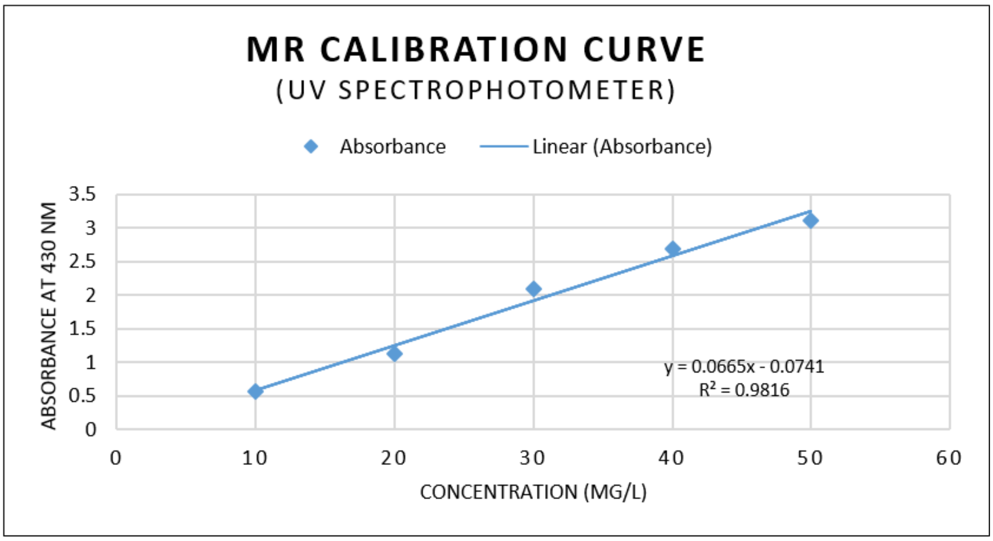

Fig. S1: MR calibration curves

Standard curves showing the linear range of MR concentrations measured by the **(a) Plate reader**, where the X-axis represents the MR concentration in mg/l and the Y-axis shows the absorbance at 450 nm, and the **(b) UV spectrophotometer,** where the X-axis represents the MR concentration in mg/l and the Y-axis shows the absorbance at 430 nm. R^2 is the square of correlation coefficient between concentration and absorbance.

Figure S2

a)

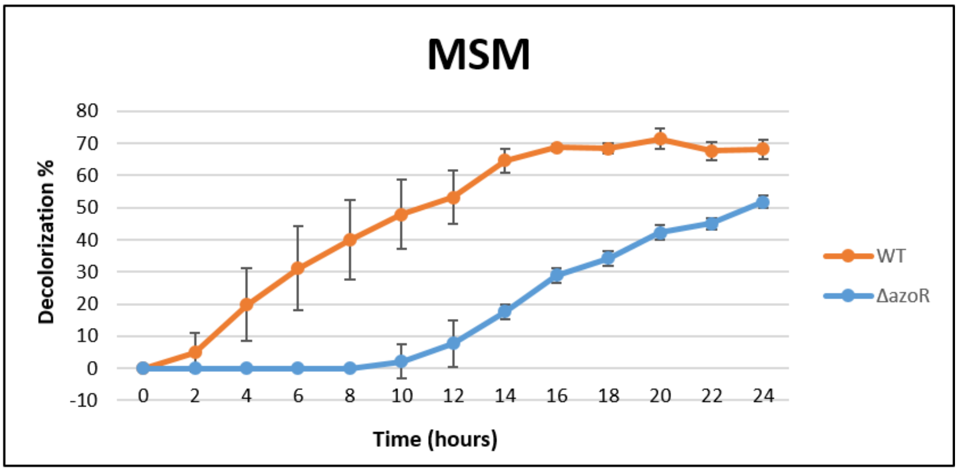

b)

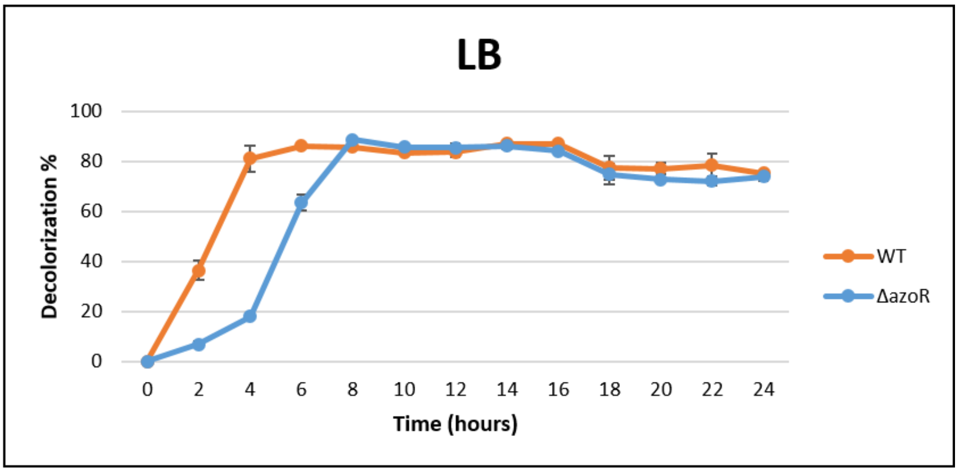

Fig. S2: Differences in decolorization of MR by the WT strain of *E. coli* K-12 and Δ*azoR*

Line graphs showing the differences in decolorization activity evident over the course of 24 hours when cultured in **(a) MSM** and **(b) LB.** The X-axis represents the time course for decolorization activity in hours, whereas the Y-axis represents the decolorization %. The orange line displays the decolorization activity of the WT *E. coli* strain and the blue line displays the decolorization activity of the Δ*azoR*. Data are the mean of three replicates and the error bars represent standard deviation.

Figure S3

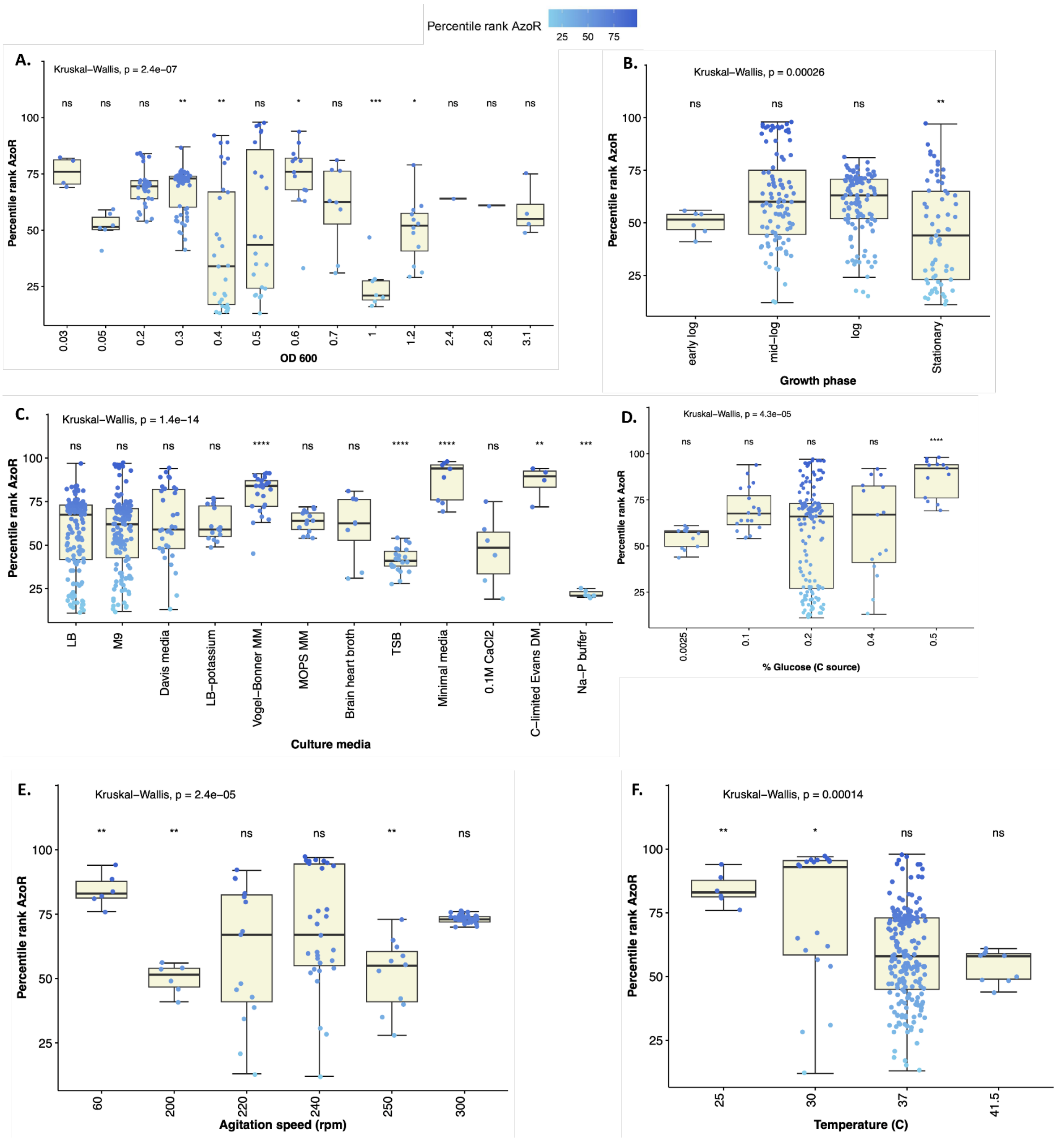

Fig. S3: Computationally predicted effects of six growth conditions on the expression of *azoR* in *E. coli* strains.

Boxplots showing the predicted effects of (A) OD_{600}, (B) Growth phase, (C) Culture media, (D) Glucose concentrations, (E) Agitation speed and (F) Temperature on *azoR* expression. The figure was generated by in the R environment with packages ggplot2 and ggpubr.

Fig. S4: Functional clustering of *azoR* co-expressed genes in at least five GEO datasets

Heatmaps displaying the clustering of genes co-expressed with *azoR* in at least five GEO datasets. Clusters **(a-h)** were generated using DAVID according to their functional annotations. The horizontal axis displays the annotated functions, while the vertical axis displays the genes grouped per cluster. Shades of green and grey denote positive and negative report of the annotated function, respectively.

Figure S4

a.

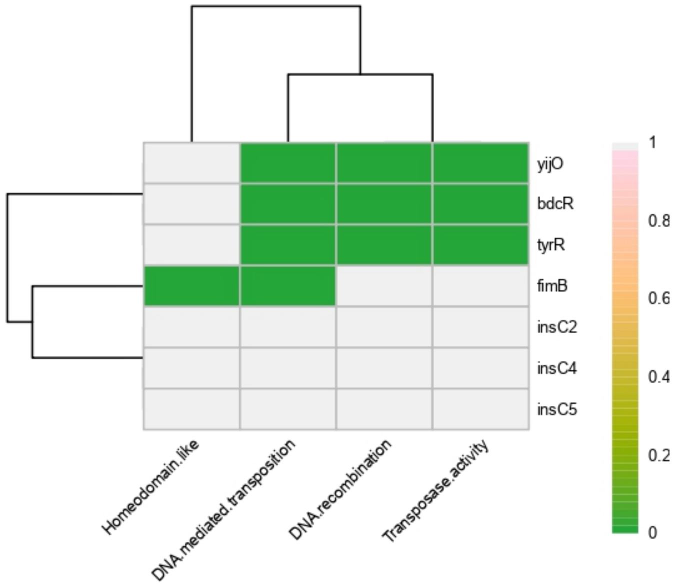
yijO
bdcR
tyrR
fimB
insC2
insC4
insC5
Homeodomain.like
DNA.mediated.transposition
DNA.recombination
Transposase.activity
1
0.8
0.6
0.4
0.2
0

b.

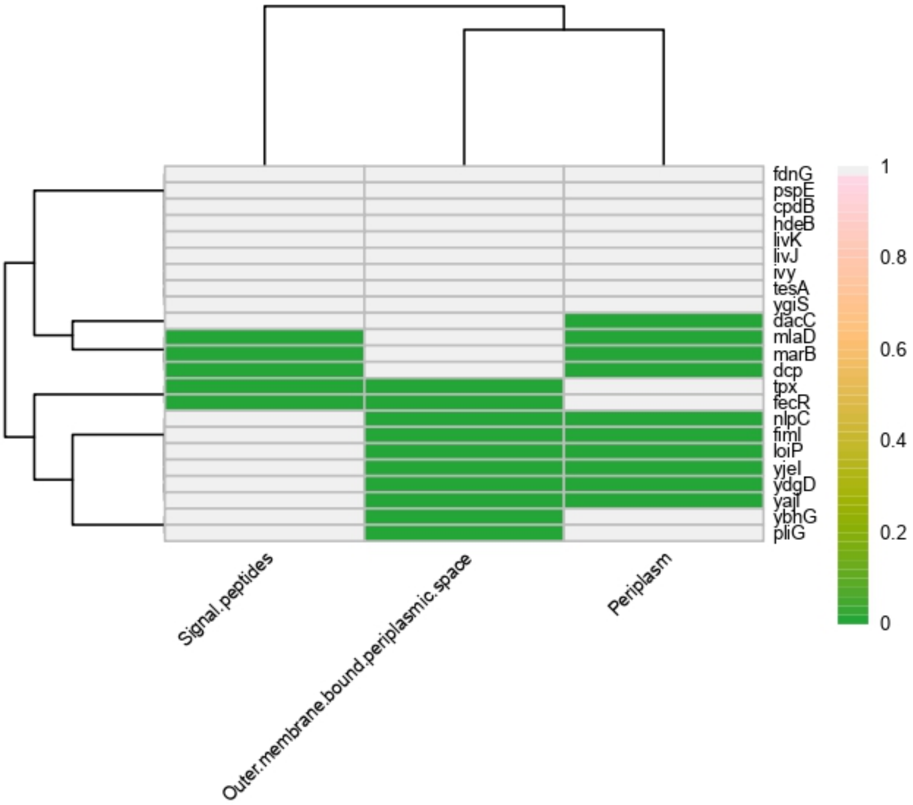
fdnG
pspE
cpdB
hdeB
livK
livJ
ivy
tesA
ygiS
dacC
mlaD
marB
dcp
tpx
fecR
nlpC
fimI
loiP
yjeI
ydgD
yajI
ybhG
pliG
Signal.peptides
Outer.membrane.bound.periplasmic.space
Periplasm
1
0.8
0.6
0.4
0.2
0

c.

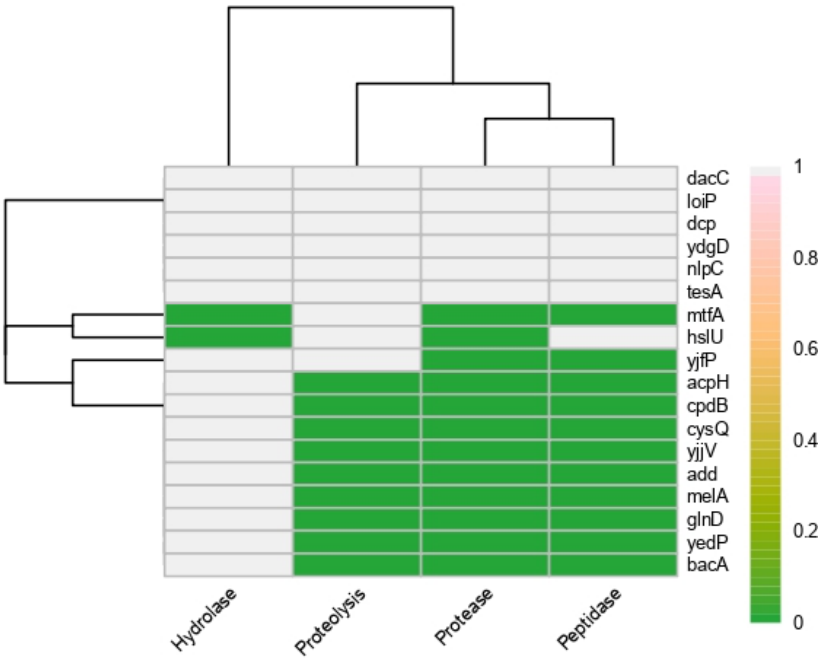
dacC
loiP
dcp
ydgD
nlpC
tesA
mtfA
hslU
yjfP
acpH
cpdB
cysQ
yjjV
add
melA
glnD
yedP
bacA
Hydrolase
Proteolysis
Protease
Peptidase
1
0.8
0.6
0.4
0.2
0

d.

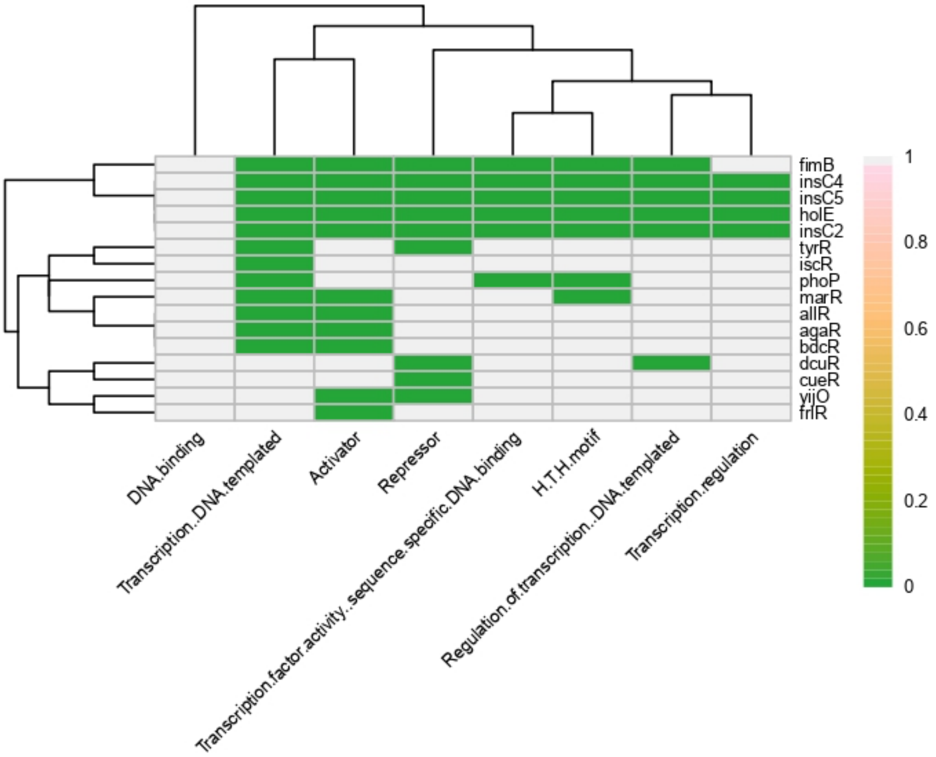
fimB
insC4
insC5
holE
insC2
tyrR
iscR
phoP
marR
allR
agaR
bdcR
dcuR
cueR
yijO
frlR
DNA.binding
Transcription..DNA.templated
Activator
Repressor
Transcription.factor.activity..sequence.specific.DNA.binding
H.T.H.motif
Regulation.of.transcription..DNA.templated
Transcription.regulation
1
0.8
0.6
0.4
0.2
0

e.

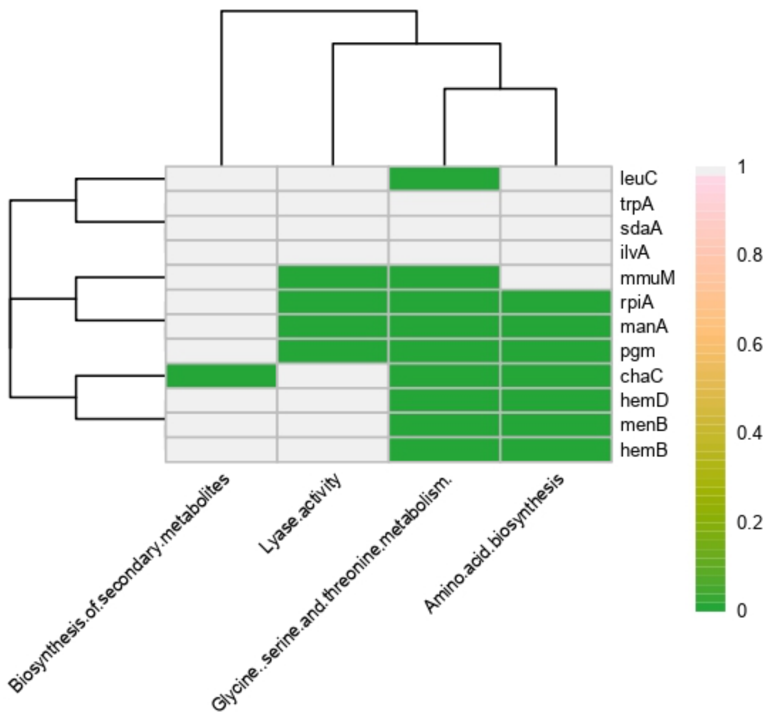
leuC
trpA
sdaA
ilvA
mmuM
rpiA
manA
pgm
chaC
hemD
menB
hemB
1
0.8
0.6
0.4
0.2
0
Biosynthesis.of.secondary.metabolites
Lyase.activity
Glycine..serine.and.threonine.metabolism.
Amino.acid.biosynthesis

f.

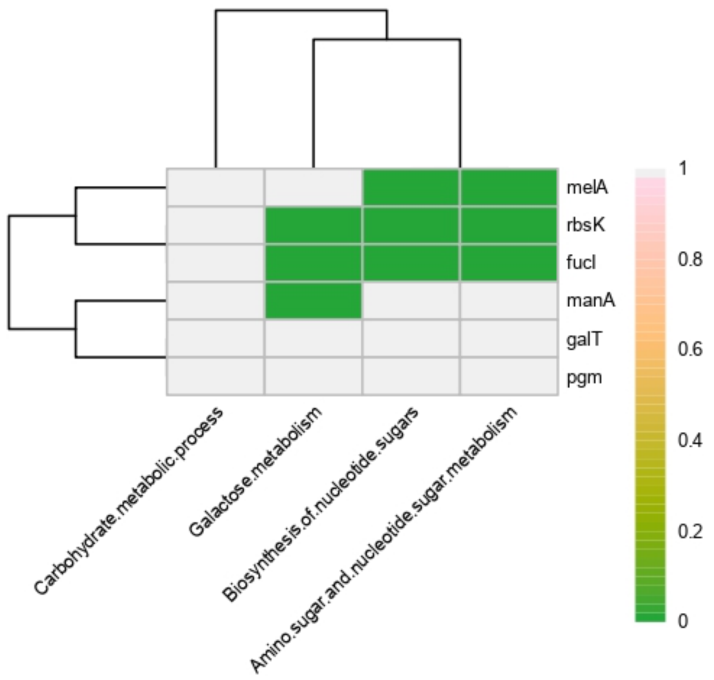
melA
rbsK
fucI
manA
galT
pgm
1
0.8
0.6
0.4
0.2
0
Carbohydrate.metabolic.process
Galactose.metabolism
Biosynthesis.of.nucleotide.sugars
Amino.sugar.and.nucleotide.sugar.metabolism

g.

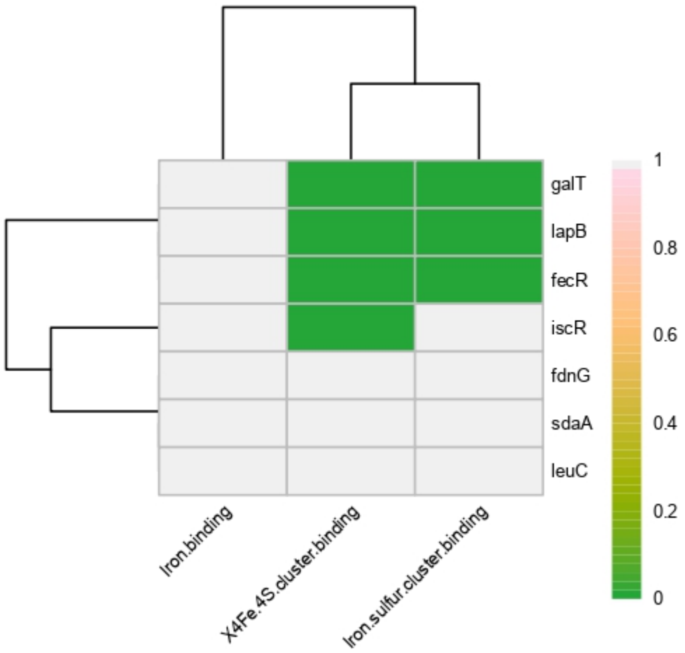
galT
lapB
fecR
iscR
fdnG
sdaA
leuC
Iron.binding
X4Fe.4S.cluster.binding
Iron.sulfur.cluster.binding
1
0.8
0.6
0.4
0.2
0

h.

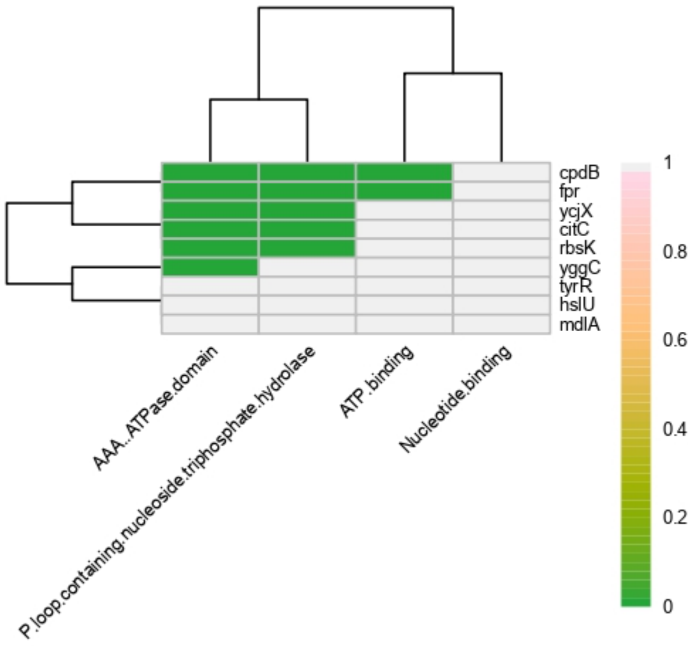
cpdB
fpr
ycjX
citC
rbsK
yggC
tyrR
hslU
mdlA
AAA..ATPase.domain
P.loop.containing.nucleoside.triphosphate.hydrolase
ATP.binding
Nucleotide.binding
1
0.8
0.6
0.4
0.2
0

Figure S5

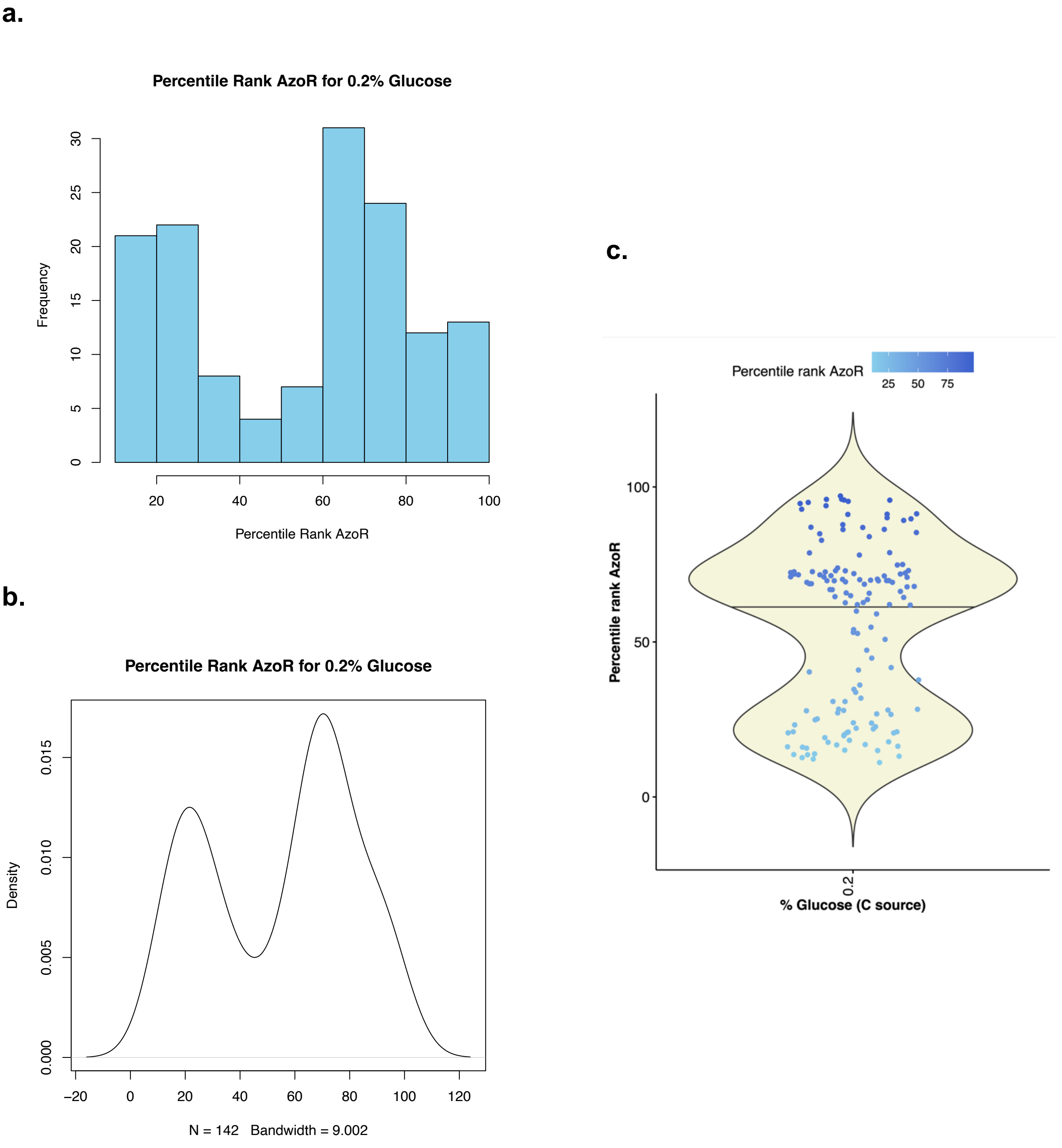

Fig. S5: Bimodal distribution of GEO-retrieved *azoR* expression data at glucose 0.2% concentrations

The large spread of *azoR* percentile ranks of gene expression data is expressed as two subpopulations (bimodal distribution) within the 142 samples grown at 0.2% glucose. These subpopulations are clearly visualized as (a) a histogram, (b) a density plot, and (c) a beanplot for the percentile rank scores of expression values.

Cited Bibliography:

[1] Lucas M, Mertens V, Corbisier A, Vanhulle S (2008) Synthetic dyes decolourisation by white-rot fungi : Development of original microtitre plate method and screening. 42:97–106. https://doi.org/10.1016/j.enzmictec.2007.07.023

[2] Mercier C, Chalansonnet V, Orenga S, Gilbert C (2013) Characteristics of major *Escherichia coli* reductases involved in aerobic nitro and azo reduction. J Appl Microbiol 115:n/a-n/a. https://doi.org/10.1111/jam.12294

[3] Zenno S, Koike H, Kumar AN, Jayaraman R, Tanokura M, Saigo K (1996) Biochemical characterization of NfsA, the *Escherichia coli* major nitroreductase exhibiting a high amino acid sequence homology to Frp, a *Vibrio harveyi* flavin oxidoreductase. J Bacteriol 178:4508–4514. https://doi.org/10.1128/jb.178.15.4508-4514.1996

[4] Paterson ES, Boucher SE, Lambert IB (2002) Regulation of the *nfsA* gene in *Escherichia coli* by SoxS. J Bacteriol 184:51–58. https://doi.org/10.1128/JB.184.1.51-58.2002

[5] Barbosa TM, Levy SB (2000) Differential expression of over 60 chromosomal genes in *Escherichia coli* by constitutive expression of MarA. J Bacteriol 182:3467–3474. https://doi.org/10.1128/JB.182.12.3467-3474.2000

[6] Ackerley DF, Gonzalez CF, Park CH, Blake R, Keyhan M, Matin A (2004) Chromate-Reducing Properties of Soluble Flavoproteins from *Pseudomonas putida* and *Escherichia coli*. Appl Environ Microbiol 70:873–882. https://doi.org/10.1128/AEM.70.2.873-882.2004

[7] Barak Y, Thorne SH, Ackerley DF, Lynch S V., Contag CH, Martin A (2006) New enzyme for reductive cancer chemotheraphy, YieF, and its improvement by directed evolution. Mol Cancer Ther 5:97–103. https://doi.org/10.1158/1535-7163.MCT-05-0365

[8] Crescente V, Holland SM, Kashyap S, Polycarpou E, Sim E, Ryan A (2016) Identification of novel members of the bacterial azoreductase family in *Pseudomonas aeruginosa*. Biochem J 473:549–558. https://doi.org/10.1042/BJ20150856

[9] Bianchi V, Reichard P, Eliasson R, et al (1993) *Escherichia coli* ferredoxin NADP+ reductase: Activation of *E. coli* anaerobic ribonucleotide reduction, cloning of the gene (*fpr*), and overexpression of the protein. J Bacteriol 175:1590–1595. https://doi.org/10.1128/jb.175.6.1590-1595.1993

[10] Kim KS, Pelton JG, Inwood WB, Andersen U, Kustu S, Wemmer DE (2010) The Rut pathway for pyrimidine degradation: Novel chemistry and toxicity problems. J Bacteriol 192:4089–4102. https://doi.org/10.1128/JB.00201-10

[11] Shimada T, Hirao K, Kori A, Yamamoto K, Ishihama A (2007) RutR is the uracil/thymine-sensing master regulator of a set of genes for synthesis and degradation of pyrimidines. Mol Microbiol 66:744–757. https://doi.org/10.1111/j.1365-2958.2007.05954.x

[12] Zimmer DP, Soupene E, Lee HL, et al (2000) Nitrogen regulatory protein C-controlled genes of *Escherichia coli*: Scavenging as a defense against nitrogen limitation. Proc Natl Acad Sci U S A 97:14674–14679. https://doi.org/10.1073/pnas.97.26.14674

[13] Baker LMS, Poole LB (2003) Catalytic Mechanism of Thiol Peroxidase from *Escherichia coli*: sulfenic acid formation and overoxidation of essential CYS61. J Biol Chem. https://doi.org/10.1074/JBC.M209888200

[14] Burstein C, Kepes A (1971) The α-galactosidase from *Escherichia coli* K12. BBA - Gen Subj 230:52–63. https://doi.org/10.1016/0304-4165(71)90053-5

[15] Ducey TF, Dyer DW (2002) Rapid identification of EZ:: TN™ transposon insertion sites in the genome of *Neisseria gonorrhoeae*. In: Epic. Forum. pp 6–7

[16] Zhou K, Zhou L, Lim Q, Zou R, Stephanopoulos G, Too HP (2011) Novel reference genes for quantifying transcriptional responses of *Escherichia coli* to protein overexpression by quantitative PCR. BMC Mol Biol 12:18. https://doi.org/10.1186/1471-2199-12-18
